# Unveiling a New Antimicrobial Peptide with Efficacy against *P. aeruginosa* and *K. pneumoniae* from Mangrove-Derived *Paenibacillus thiaminolyticus* NNS5-6 and Genomic Analysis

**DOI:** 10.3390/antibiotics13090846

**Published:** 2024-09-05

**Authors:** Namfa Sermkaew, Apichart Atipairin, Sucheewin Krobthong, Chanat Aonbangkhen, Yodying Yingchutrakul, Jumpei Uchiyama, Nuttapon Songnaka

**Affiliations:** 1 School of Pharmacy, Walailak University, Thasala, Nakhon Si Thammarat 80160, Thailand; namfa.se@wu.ac.th (N.S.); apichart.at@wu.ac.th (A.A.); 2 Drug and Cosmetics Excellence Center, Walailak University, Thasala, Nakhon Si Thammarat 80160, Thailand; 3 Center of Excellence in Natural Products Chemistry (CENP), Department of Chemistry, Faculty of Science, Chulalongkorn University, Bangkok 10330, Thailand; sucheewin.k@chula.ac.th (S.K.); chanat.a@chula.ac.th (C.A.); 4 Center of Excellence on Petrochemical and Materials Technology, Chulalongkorn University, Bangkok 10330, Thailand; 5 National Center for Genetic Engineering and Biotechnology, National Science and Technology Development Agency, Pathum Thani 12120, Thailand; yodying.yin@biotec.or.th; 6 Department of Bacteriology, Graduate School of Medicine, Dentistry and Pharmaceutical Sciences, Okayama University, Okayama 700-8558, Japan; uchiyama@okayama-u.ac.jp

**Keywords:** antimicrobial peptide, antimicrobial resistance, bacterial genome, biosynthetic gene cluster, *Klebsiella pneumoniae*, Mangrove, mass spectrometry, NNS5-6, *Paenibacillus thiaminolyticus*, *Pseudomonas aeruginosa*

## Abstract

This study focused on the discovery of the antimicrobial peptide (AMP) derived from mangrove bacteria. The most promising isolate, NNS5-6, showed the closest taxonomic relation to *Paenibacillus thiaminolyticus*, with the highest similarity of 74.9%. The AMP produced by *Paenibacillus thiaminolyticus* NNS5-6 exhibited antibacterial activity against various Gram-negative pathogens, especially *Pseudomonas aeruginosa* and *Klebsiella pneumoniae*. The peptide sequence consisted of 13 amino acids and was elucidated as Val-Lys-Gly-Asp-Gly-Gly-Pro-Gly-Thr-Val-Tyr-Thr-Met. The AMP mainly exhibited random coil and antiparallel beta-sheet structures. The stability study indicated that this AMP was tolerant of various conditions, including proteolytic enzymes, pH (1.2–14), surfactants, and temperatures up to 40 °C for 12 h. The AMP demonstrated 4 µg/mL of MIC and 4–8 µg/mL of MBC against both pathogens. Time-kill kinetics showed that the AMP acted in a time- and concentration-dependent manner. A cell permeability assay and scanning electron microscopy revealed that the AMP exerted the mode of action by disrupting bacterial membranes. Additionally, nineteen biosynthetic gene clusters of secondary metabolites were identified in the genome. NNS5-6 was susceptible to various commonly used antibiotics supporting the primary safety requirement. The findings of this research could pave the way for new therapeutic approaches in combating antibiotic-resistant pathogens.

## 1. Introduction

Antimicrobial resistance (AMR) poses a significant challenge to global public health. Its severity is exacerbated by the overuse and inappropriate application of antimicrobial agents in humans, animals, and plants. This contributes to an increase in new resistant microorganisms in the environment. Presently, AMR results in over 700,000 deaths annually worldwide, creating substantial economic and health impacts [1]. The “ESKAPE” pathogens a group that includes *Enterococcus faecium*, *Staphylococcus aureus*, *Klebsiella pneumoniae*, *Acinetobacter baumannii*, *Pseudomonas aeruginosa*, and *Enterobacter* species [2]. This group is categorized as “critical” on the World Health Organization’s (WHO) priority list of bacterial pathogens, underscoring the urgent need for novel antibiotics to address drug resistance. *Pseudomonas aeruginosa*, a ubiquitous Gram-negative bacterium, is widely recognized as an opportunistic pathogen notorious for its antibiotic resistance. It is a leading cause of nosocomial infections and ventilator-associated pneumonia, presenting a substantial challenge in clinical settings because of its ability to form biofilms, which complicates treatment [3,4]. *P. aeruginosa* contributes to more than 5% of infectious exacerbations in patients with chronic obstructive pulmonary disease (COPD) and is associated with elevated mortality rates among these individuals [3]. Similarly, *Klebsiella pneumoniae* has emerged as a virulent pathogen, attributable to the rising number of severe infections. Given the evolutionary diversity among clinical strains, it plays a significant role in various infection models, including pneumonia, liver abscesses, and gastrointestinal tract colonization [5]. Exploring new antimicrobial agents is critical in addressing the persistent threat of infectious diseases caused by *P. aeruginosa* and *K. pneumoniae*.

Microorganisms, especially bacteria, are well-suited for the large-scale synthesis of bioactive compounds. Since the discovery of penicillin, microbial secondary metabolites have been a primary source of novel antimicrobial agents. Marine bacteria have emerged as promising sources of therapeutic compounds, including antibacterial, antiviral, antifungal, and anticancer agents [6]. The mangrove ecosystem, characterized by harsh environmental conditions such as high tides, hypersaline waters, and large temperature fluctuations, provides a unique habitat that fosters the production of bioactive compounds [7,8]. Genomic studies suggest that bacteria from harsh environments, with their often-large genomes, have the potential to produce a diverse array of secondary metabolites, surpassing previous estimates. This highlights the importance of exploring these unique habitats for novel antimicrobial compounds [9]. Attention has been focused on antimicrobial peptides (AMPs), which have been investigated for the treatment of infections [10]. AMPs are an essential component of the innate immune system present in all living organisms, playing a crucial role as the frontline defense against pathogens. They vary in the length of their amino acid residues and are composed of charged and hydrophobic amino acids. In general, AMPs are unstructured and potentially form amphipathic alpha-helical or beta-sheet structures in bacterial cell membranes. These peptides disrupt membranes without target–receptor specificity and have high-affinity binding interactions; therefore, they are less likely to induce resistance in pathogens [11].

The objective of this study was to isolate mangrove bacteria capable of producing an AMP effective against *P. aeruginosa* and *K. pneumoniae*. The amino acid sequence and properties of the AMP were characterized. The genomic information of the promising bacterial isolate was explored, including the biosynthetic gene clusters for secondary metabolites. This research seeks to identify new antimicrobial resources and provide valuable insights to develop alternative agents for facing the emergence of antimicrobial-resistant infections.

## 2. Results

### 2.1. Antimicrobial Investigation of Bacterial Isolates from Mangrove Sediments

Mangrove sediments were collected from five different locations at Banlaem Mangrove, Thasala District, Nakhon Si Thammarat Province, Thailand. The pH of the sediments ranged from 6.26 to 8.00, and the salinity was between 9.00 and 10.00 ppt. Colonies grown on Mueller Hinton (MH) agar, Zobell Marine (ZM) agar, and Starch Casein (SC) agar were screened for antibacterial activities against *P. aeruginosa* TISTR 357 using the soft agar overlay technique. The antibacterial activity of the isolates was expanded to include activity against various bacterial pathogens. Only one isolate, NNS5-6, cultured on MH agar, exhibited antibacterial activity against *P. aeruginosa* TISTR 357. The cell-free supernatant (CFS) collected from a 24-h preculture of NNS5-6 in MH broth showed an expanded antibacterial spectrum against Gram-negative bacteria, including *P. aeruginosa* TISTR 357, *K. pneumoniae* TISTR 1383, *Escherichia coli (E. coli)* TISTR 887, *Salmonella typhimurium (S. typhimurium)* TISTR 1469, and *Vibrio parahaemolyticus (V. parahaemolyticus)* TISTR 1596, with inhibition zones ranging from 12.70 ± 0.25 to 14.84 ± 0.15 mm. In contrast, there was no activity against Gram-positive bacteria, such as *Staphylococcus aureus (S. aureus)* TISTR 517 and methicillin-resistant *Staphylococcus aureus* (MRSA) strain 2468 (Table 1). The standard antibiotics vancomycin and colistin were used as positive controls to compare the degree of antibacterial activity of the active isolate. Therefore, the NNS5-6 isolate was selected for further antibacterial studies.

### 2.2. Production Kinetics of Antibacterial Compounds of NNS5-6

**Figure 1:**
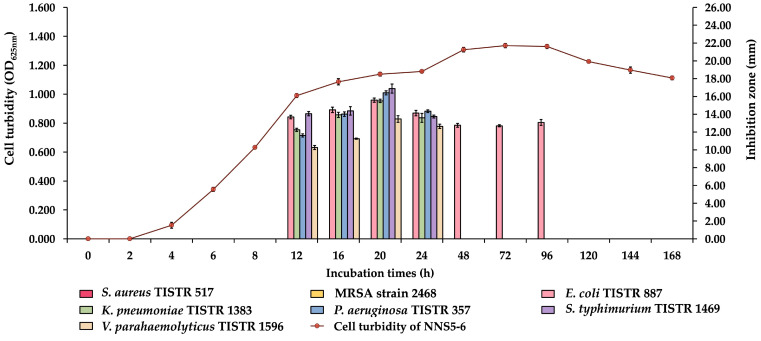
The growth profile of NNS5-6 and its production kinetics of antibacterial components were determined during the incubation periods. The growth curve was reported by the turbidity of the cell suspension (primary *y*-axis). The antibacterial activity against bacterial pathogens is presented by the inhibition zones (secondary *y*-axis).

This study investigated the production of antibacterial compounds by NNS5-6 over the incubation period with culture growth monitored through cell suspension turbidity. The antibacterial activity of the collected CFS at different incubation times was determined by measuring inhibition zones against various bacterial pathogens. The antibacterial activity was observed during the early stationary phase of the growth curve. The results revealed that the initial and maximum activity of the antibacterial compounds production by NNS5-6 was found at 12 h and 20 h of incubation, respectively. The maximum inhibition zone of NNS5-6 CFS at 20 h was in the range of 15.50 ± 0.20 to 16.89 ± 0.50 mm. The antibacterial activity declined after 20 h of incubation until it diminished at 24 h during the stationary phase of growth, except in E. coli TISTR 887, where the antibacterial activity of NNS5-6 CFS remained until 96 h of incubation. In contrast, the Gram-positive bacteria, S. aureus TISTR 517 and MRSA strain 2468, were not inhibited by NNS5-6 CFS at any incubation period. NNS5-6 demonstrated the ability to produce antibacterial compounds against Gram-negative bacteria within 20 h of incubation (Figure 1).

### 2.3. Purification of Antibacterial Compounds of NNS5-6

The antibacterial components produced by a twenty-hour-old NNS5-6 culture were purified through sequential steps, including ammonium sulfate precipitation, cation-exchange chromatography, and size-exclusion chromatography. The fractions obtained at each step were tested for antibacterial activity against *P. aeruginosa* TISTR 357 using the agar well diffusion assay. A bioassay-guided approach was used to identify the fraction with antibacterial activity (Figure S1a). The active fraction was collected to determine the presence of the active peptide band, and the purity of the purified peptide was confirmed using a 15% gel of SDS-PAGE. The SDS-PAGE analysis revealed a single stained protein band in Lane 1 of the half-excised gel, with a molecular weight below approximately 5 kDa. The same location of the inhibition zone was observed in the other half-excised gel overlaid with *P. aeruginosa* TISTR 357, demonstrating the effectiveness of the purification procedures (Figure S1b). The efficiency of these procedures was assessed using a purification balance sheet. The specific activity increased with each purification step, indicating the effectiveness of the procedures in purifying the active peptide. The final purification step resulted in a 13.17-fold increase in purification power and yielded 10.55% of the active peptide compared with the initial crude product (Table 2).

### 2.4. De Novo Amino Acid Sequence of the Purified AMP and Determination of Its Secondary Structure

**Figure 2:**
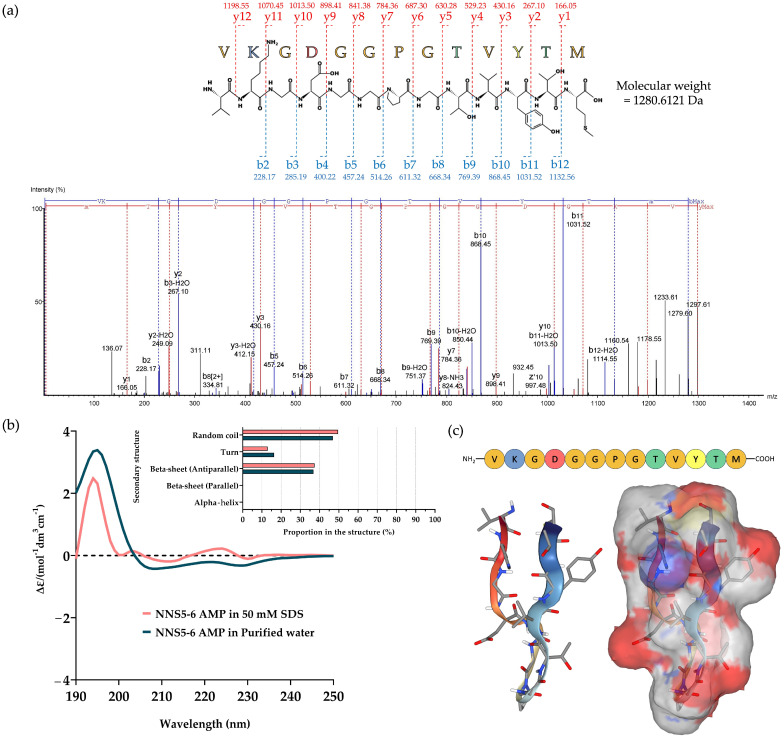
The amino acid sequence of the purified AMP was elucidated by de novo amino acid sequencing using a molecular fragmentation technique (dashed blue line and dashed red line indicate b-ion and y-ion, respectively) from mass spectrometry (**a**). The secondary structure of NNS5-6 AMP was determined by CD spectroscopy. The experiment was designed to compare the structural changes in the dissolved AMP in purified water and 50 mM SDS. The proportion of secondary structures was analyzed by BeStSel and compared in different solvents (**b**). The 3D molecular model of NNS5-6 AMP was predicted using PEP-FOLD4. The beta-sheet structure of the AMP is shown in a cartoon style. The molecular surface reveals the hydrophobic region represented by grey and the hydrophilic regions with negative and positive electrostatic potentials represented by red and blue, respectively (**c**).

The purified AMP of NNS5-6 was subjected to amino acid sequencing using tandem mass spectrometry. The molecular weight of the peptide was determined by mass spectrometry. The peptide fragmentation detected the parent molecule having a mass of 1297.61 Da in the positive ion mode. The de novo algorithm was used to predict the amino acid sequence from b-ion and y-ion fragmentations. The AMP, composed of 13 amino acid residues, was sequenced as Val-Lys-Gly-Asp-Gly-Gly-Pro-Gly-Thr-Val-Tyr-Thr-Met, with an average local confidence (ALC) score of 75% (Figure 2a). The results of de novo sequencing indicated that the final amino acid, methionine, was oxidized with an incorporated oxygen atom. Therefore, the molecular weight of the peptide was 1280.6121 Da. The physicochemical properties of the peptide were predicted using ProtParam on the Expasy server (https://web.expasy.org/protparam/, accessed on 10 July 2024). The analysis revealed a theoretical pI of 5.81. The peptide contained one positively charged amino acid, lysine, and one negatively charged residue, aspartic acid. The predicted net charge was 0 at pH 7.4. The grand average of hydropathy (GRAVY) value of the peptide was calculated to be −0.231, predicted by hydrophobic amino acids valine, proline, and methionine, as well as hydrophilic amino acids lysine, glycine, aspartic acid, threonine, and tyrosine.

Circular dichroism (CD) spectroscopy was used to determine the secondary structure of NNS5-6 AMP in different solvents. Purified water was used to study its native conformation, while 50 mM SDS, above the critical micelle concentration, was employed to simulate the negatively charged environment of bacterial cell membranes for studying AMP interactions. The CD spectra of NNS5-6 AMP showed similar delta epsilon in both purified water and 50 mM SDS solution. The CD spectra scanned in the range of 190–240 nm revealed a positive band with a magnitude at 195 nm and a negative band with a magnitude at 215 nm in both solvent systems. The CD spectra indicated a beta-sheet conformation of the peptide in both solvents. In addition, the negative band at 200–210 nm suggested the presence of a random coil structure. The secondary structure components of the NNS5-6 AMP were calculated using CD spectra via the BeStSel web-based service. In purified water, the NNS5-6 AMP showed structural components of 46.9% random coil conformation, 36.8% antiparallel beta-sheet, and 16.3% turn conformation. A slight difference in secondary structure components was observed when NNS5-6 AMP was dissolved in the SDS micelle environment. The major component remained as a random coil at 49.6%, while the beta-sheet (antiparallel) structure was 37.4%, and the turn conformation was 13.0% (Figure 2b). The findings indicated that the NNS5-6 AMP primarily exhibited a beta-sheet (antiparallel) secondary structure with a random coil conformation. In the presence of SDS micelles, the interaction between NNS5-6 AMP and SDS micelles slightly altered the secondary structure components of the AMP but did not change the overall type of secondary structures compared with the native environment. The 3D molecular model further supported the analyzed CD spectra results. The NNS5-6 AMP displayed the beta-sheet conformation with an antiparallel orientation, with a turn at the proline amino acid and a random coil at the N- and C-termini. The predicted molecular surface revealed the hydrophobic and hydrophilic regions of NNS5-6 AMP (Figure 2c). The results suggest that the NNS5-6 AMP likely maintains a stable antiparallel beta-sheet structure at membrane surfaces, supporting the hypothesis that the cationic side of the AMP first interacts electrostatically with the anionic membrane surface and destabilizes the lipid bilayer of the cell membrane with the hydrophobic part of the peptide [12].

### 2.5. Investigation of the Antibacterial Activities of the AMP

The antibacterial activities of the NNS5-6 AMP against *P. aeruginosa* TISTR 357 and *K. pneumoniae* TISTR 1383 were studied using a microdilution assay, with colistin as the standard. Both *P. aeruginosa* TISTR 357 and *K. pneumoniae* TISTR 1383 were inhibited by NNS5-6 AMP at the same MIC of 4 µg/mL, compared with an MIC of 1 µg/mL for colistin. However, the concentrations required for the bactericidal effect of NNS5-6 AMP differed between the two pathogens. The bactericidal activity of NNS5-6 AMP required a concentration with a two-fold higher MIC concentration for killing *K. pneumoniae* TISTR 1383, while for *P. aeruginosa* TISTR 357, the bactericidal concentration was equal to the MIC. Colistin exhibited bactericidal activity at the MIC for both bacterial pathogens (Table 3).

### 2.6. The Antibacterial Activity of NNS5-6 Derived AMP on Bacterial Pathogens Observed Using Scanning Electron Microscopy (SEM) Analysis

**Figure 3:**
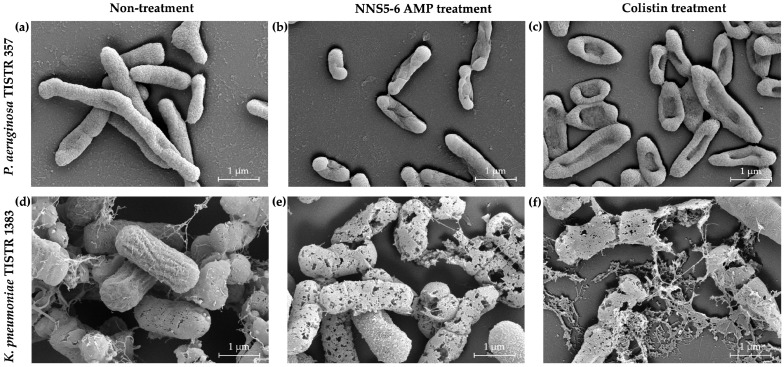
The effects of NNS5-6 AMP and colistin on *P. aeruginosa* TISTR 357 and *K. pneumoniae* TISTR 1383 were observed as morphological changes visualized under a scanning electron microscope at 20,000× magnification. The untreated condition of both bacteria was incubated with 0.85% NaCl, *P. aeruginosa* TISTR 357 (**a**) and *K. pneumoniae* TISTR 1383 (**d**). Effects of 1× MIC of NNS5-6 AMP on *P. aeruginosa* TISTR 357 (**b**) and *K. pneumoniae* TISTR 1383 (**e**). Effects of 1× MIC of colistin on *P. aeruginosa* TISTR 357 (**c**) and *K. pneumoniae* TISTR 1383 (**f**).

Alterations in cell morphology and membrane integrity of *P. aeruginosa* TISTR 357 and *K. pneumoniae* TISTR 1383 treated with antibacterial compounds were observed using SEM. The NNS5-6 AMP and colistin demonstrated antibacterial activity against these bacterial pathogens at 1× MIC. The cell surfaces of both pathogens responded differently to the antibacterial compounds. In untreated *P. aeruginosa*, the intact cell membrane retained its original rod shape with a smooth surface, indicating membrane integrity (Figure 3a). NNS5-6 AMP caused the *P. aeruginosa* cells to develop a rough surface and shrink. The center region of the treated cells appeared intruded, indicating cell membrane breakage and the release of cytoplasm after the cell ruptured (Figure 3b). The action of colistin is well-known for its antibacterial activity through membrane disruption. Colistin-treated *P. aeruginosa* cells exhibited a killing effect, observed as holes at the center of the cells and deflated cells, confirming membrane disruption (Figure 3c). For *K. pneumoniae*, untreated cells maintained their shape and showed some exopolysaccharide deposits around them, indicating vigorous growth (Figure 3d). Upon treatment with NNS5-6 AMP, *K. pneumoniae* cells exhibited signs of rupture and cytoplasmic leakage, characterized by a porous morphology (Figure 3e). Similarly, colistin-treated cells displayed membrane damage, leading to pore formation (Figure 3f). The observed differences in morphological changes between treated *P. aeruginosa* cells and *K. pneumoniae* compared with untreated controls highlight the distinct membrane disruption characteristics induced by NNS5-6 AMP and colistin. This investigation suggests that NNS5-6 AMP effectively targets and damages bacterial membranes.

### 2.7. Time-Kill Assay of NNS5-6 AMP

**Figure 4:**
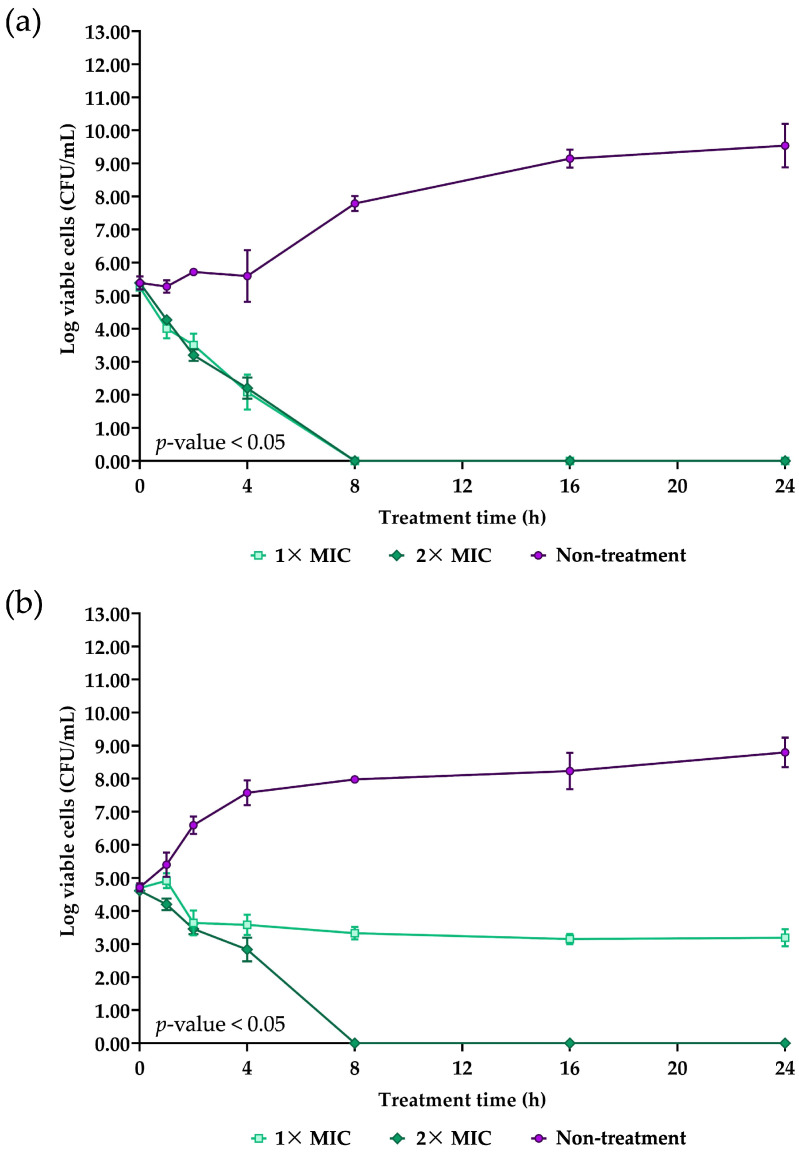
The time-kill assay was performed to determine the time- and concentration-dependent effects of NNS5-6 AMP. The experiments used 1× and 2× MIC of NNS5-6 AMP for *P. aeruginosa* TISTR 357 (**a**) and *K. pneumoniae* TISTR 1383 (**b**). The efficacy of the AMP was monitored by counting viable cells during the treatment period of up to 24 h. The experiment was performed in triplicate, and the log of viable cells is expressed as the mean with standard deviation (SD). The statistical significance between different concentrations in treatment and non-treatment conditions was determined using two-way ANOVA (*p*-value < 0.05).

The time-kill assay was conducted to determine the mode of antibacterial action of NNS5-6 AMP. The NNS5-6 AMP exhibited different inhibitory effects on *P. aeruginosa* TISTR 357 compared with *K. pneumoniae* TISTR 1383. For *P. aeruginosa* TISTR 357, a significant reduction in viable cells was observed within the first hour of treatment with 1× MIC and 2× MIC of NNS5-6 AMP (Figure 4a). The eradication effect of NNS5-6 AMP on viable cells was achieved when treatment time reached 8 h with concentrations of 1× and 2× MIC. From the initial treatment until the viable cells were eradicated, the reduction rate of viable cells was similar for 1× MIC (0.6297 log CFU/h) and 2× MIC (0.6421 log CFU/h). In contrast, *K. pneumoniae* TISTR 1383 showed a delayed response, with bacterial suppression beginning 2 h after treatment with 1× MIC. The reduction rate of viable cells was slower with 1× MIC (0.05790 log CFU/h) compared with 2× MIC (0.5715 log CFU/h). In addition, the eradication effect was observed at different concentrations of NNS5-6 AMP. The treatment with 2× MIC achieved eradication of viable cells within 8 h, whereas 1× MIC only suppressed cell growth and reduced viable cells by up to 2 log (CFU/mL) until 24 h of treatment (Figure 4b). The differences in antibacterial activity observed in the time-kill assay were consistent with the results from the microdilution assay and SEM micrography, both of which demonstrated the eradication effect. The killing curve for *K. pneumoniae* TISTR 1383 was both concentration- and time-dependent, whereas, for *P. aeruginosa* TISTR 357, it was time-dependent.

### 2.8. Studies of Cell Permeability

**Figure 5:**
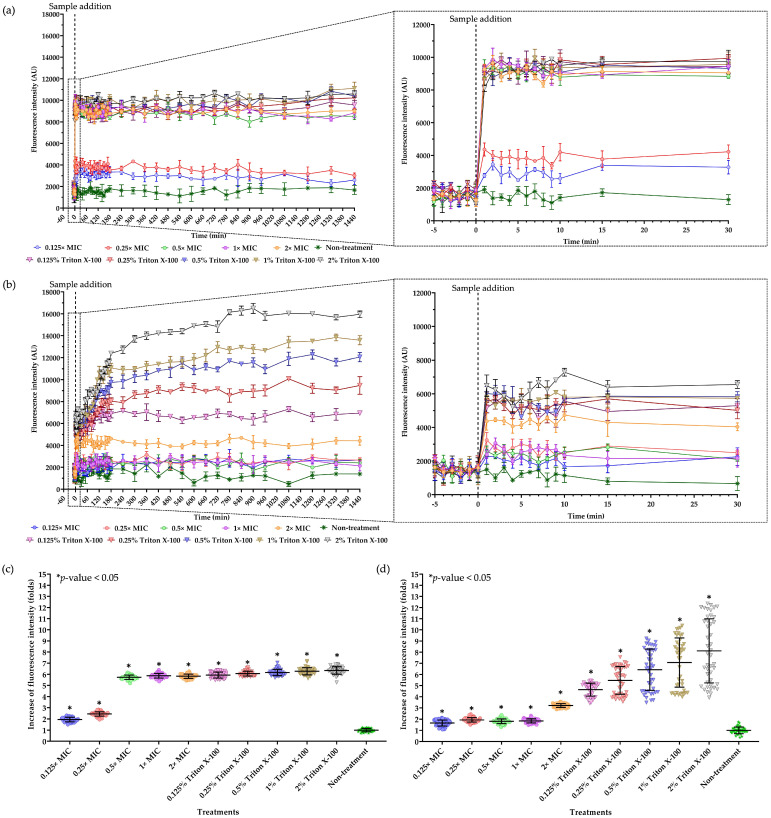
The Sytox Green uptake assay of NNS5-6 AMP-treated bacterial cells demonstrated the effect of NNS5-6 AMP on cell permeability. Cell cultures of *P. aeruginosa* TISTR 357 (**a**) and *K. pneumoniae* TISTR 1383 (**b**) were incubated with different concentrations of AMP (0.125×, 0.25×, 0.5×, 1×, and 2× MIC). Various concentrations of Triton X-100 (0.125%, 0.25%, 0.5%, 1%, and 2% *w*/*v*) were used as positive controls to indicate the levels of cell membrane permeability. Membrane permeabilization of bacterial cells was monitored by the increase in fluorescence intensity caused by the Sytox Green-DNA complex. The fluorescence intensity was observed over 24 h. The inset graph provides an expanded view of the fluorescence baseline before sample addition and the fluorescence intensity in the initial 30 min after sample addition. The increase in fluorescence intensity during the entire treatment period was expressed in folds, comparing different concentrations of samples to the non-treatment condition of *P. aeruginosa* TISTR 357 (**c**) and *K. pneumoniae* TISTR 1383 (**d**). The experiments were conducted in triplicate, with the mean and standard deviation presented. The statistical analysis was performed to compare the overall time points between treatments and non-treatments using one-way ANOVA (*p*-value < 0.05) shown in (**c**,**d**).

The Sytox Green uptake assay was used to investigate cell permeabilization of bacterial pathogens after treatment with the AMP. The loss of cell membrane function due to the AMP disturbance was monitored by the fluorescence intensity, which reflects the binding of Sytox Green to DNA as a result of membrane malfunction [13]. For *P. aeruginosa* TISTR 357, the addition of NNS5-6 AMP caused an immediate increase in fluorescence intensity. Fluorescence was found to have a plateau characteristic until 24 h of treatment. Different levels of fluorescence intensity were observed at different concentrations of NNS5-6 AMP (0.125× MIC to 2× MIC) and Triton X-100 (0.125% to 2% *w*/*v*). Higher fluorescence intensity was observed with NNS5-6 AMP at concentrations of 0.5×, 1×, and 2× MIC compared with lower concentrations of 0.25× and 0.125× MIC. Additionally, the fluorescence intensity of NNS5-6 AMP treatment at concentrations of 0.5×, 1×, and 2× MIC showed no significant differences and was comparable with the fluorescence intensity observed with Triton X-100 treatment in the concentration range of 0.125% to 0.5% (Figure 5a). The fold increase in fluorescence intensity caused by NNS5-6 AMP treatment compared with non-treatment ranged from 1.95 ± 0.28 to 5.87 ± 0.31 when using 0.125× to 2× MIC of NNS5-6 AMP, while 5.93 ± 0.34 to 6.34 ± 0.39 was observed with 0.125% to 2% Triton X-100 treatment (Figure 5c). The increased cell permeability due to NNS5-6 AMP and Triton X-100 treatment against *P. aeruginosa* TISTR 357 can be attributed to a concentration-dependent action. However, the increased fluorescence intensity distinguished the degree of permeability into two groups, with one group including concentrations of 0.5×, 1×, and 2× MIC, and the other group including 0.25× and 0.125× MIC. The 0.25× MIC was identified as the cut-off concentration for the different degrees of cell permeability caused by NNS5-6 AMP treatment in *P. aeruginosa* TISTR 357. The different permeability was observed in *K. pneumoniae* TISTR 1383 treated with NNS5-6 AMP and Triton X-100. The concentration-dependent manner was found in both NNS5-6 AMP and Triton X-100 (Figure 5b). The similar levels of fluorescence intensity in the range of 0.125×, 0.25×, 0.5×, and 1× MIC of NNS5-6 AMP showed 1.66 ± 0.37 to 1.94 ± 0.29-fold increase. Moreover, 2× MIC of NNS5-6 AMP treatment resulted in a distinguishable and the highest fluorescence intensity, with a 3.21 ± 0.29-fold increase compared with other concentrations. Triton X-100 showed increases of 4.64 ± 0.62 to 8.12 ± 2.86-fold in fluorescence intensity, which was higher than those observed with NNS5-6 AMP for the treatment of *K. pneumoniae* TISTR 1383 (Figure 5d). These findings suggest that increased cell membrane permeability due to NNS5-6 AMP is concentration-dependent in both *P. aeruginosa* TISTR 357 and *K. pneumoniae* TISTR 1383. Although the cell permeability measurement during the treatment could not distinguish between live and dead cells, the results from the Sytox Green uptake assay support the concentration- and time-dependent effects observed in the time-kill assay. These results confirm the eradication of viable cells through membrane disruption, as captured by SEM micrography.

### 2.9. Stability Studies of NNS5-6 AMP under Various Conditions

The stability of NNS5-6 AMP was evaluated under various conditions (Table 4). The antibacterial activity of NNS5-6 AMP against *P. aeruginosa* TISTR 357 was used as the primary indicator of its stability. The AMP demonstrated thermostability at temperatures up to 40 °C for 12 h (99.07 ± 0.72% to 99.80 ± 0.94% residual activity). However, at 50 °C, the activity of AMP was reduced by almost half within 1 h and was completely lost after 6 h. Furthermore, the antibacterial efficacy of NNS5-6 AMP was entirely lost when exposed to 60 °C, 80 °C, and 100 °C for 1 h and under autoclave conditions. The residual activity of the AMP was significantly reduced when incubated with proteinase K from 1 h to 12 h (83.62 ± 1.34% to 96.08 ± 3.41% residual activity). The AMP was stable when incubated with trypsin for up to 6 h (99.38 ± 1.99% residual activity). Additionally, the AMP maintained its activity for up to 12 h when incubated with α-chymotrypsin, indicating its stability under α-chymotrypsin incubation (98.46 ± 0.88% to 99.42 ± 1.22% residual activity). The results indicated the proteinaceous nature of the AMP, making it susceptible to digestion by proteinase K and trypsin while not being digested by α-chymotrypsin. The overall residual activity after digestion with proteinase K, trypsin, and α-chymotrypsin was up to 80%.

Surfactants above the critical micelle concentration were used to assess their interference with NNS5-6 AMP activity. The combination of the AMP with SDS or Triton X-100 resulted in increased antibacterial activity compared with the AMP alone. However, SDS or Triton X-100 alone showed higher antibacterial activity (121.76 ± 2.42% to 126.83 ± 2.72% residual activity) compared with the AMP combined with these surfactants (116.17 ± 0.60% to 122.52 ± 1.86% residual activity) and the AMP alone (100.00 ± 0.60% to 100.00 ± 0.93% residual activity). The results from combining the AMP with surfactants at a concentration above the critical micelle concentration suggested that interactions between NNS5-6 AMP and surfactants occurred, but further studies are needed to understand these interactions fully. The activity of the AMP after incubation at pH 1.2 was significantly lower than that of non-treated AMP at all time points (81.14 ± 1.71% to 89.62 ± 1.85% residual activity). In contrast, the activity of AMP remained above 95% when treated in a pH range of 4.5 to 10.0 (95.62 ± 1.19% to 99.23 ± 1.15% residual activity). The activity of AMP decreased in a pH- and time-dependent manner in a pH range of 12–14 (94.31 ± 0.68% to 79.05 ± 1.75% residual activity). These findings indicated that the AMP was less tolerant to extremely acidic and alkaline environments but retained activity in the pH range of 4.5–10.0. Overall, NNS5-6 AMP exhibited a stable profile against proteolytic enzymes, surfactants, and pH, with up to 80% residual activity over 12 h of treatment.

### 2.10. Phenotypic Characterization of NNS5-6

A single colony of NNS5-6 appeared as a circular, cream-colored colony with an undulating margin and a smooth surface (Figure S2a). The bacterial cells were observed under a light microscope at 1000× magnification. The one-day-old vegetative cells appeared Gram-positive-stained and rod-shaped bacilli (Figure S2b). Three-day-old endospores were oval-shaped, as confirmed by malachite green staining (Figure S2c). High-resolution SEM images showed rod-shaped bacilli with dimensions of 0.4–0.5 µm in diameter and 2.0–2.3 µm in length (Figure S2d). The endospores had ridge-like characteristics with dimensions of 0.8–1.0 µm in diameter and 1.8–2.0 µm in length (Figure S2e). The light microscope and SEM images provided consistent morphological results for both vegetative cells and spores. The reduction in cell dimensions from vegetative cells to spores can be attributed to the dormant process, which helps the species tolerate stressful environments and enhances survival adaptability.

### 2.11. Genome Insight for Coding Sequence Annotation and Whole-Genome Phylogenetic Analysis

The read quality was high, and the assembled genome had a size of 6,522,808 bp with a 139× sequencing depth, constructed by de novo assembly using the Velvet version 1.2.10. The assembled genome contained 283 contigs with an average contig length of 74,721 bp. The GC content was 53.20%. The genome presented a completeness of 99.68%. The genome assembly had 0.82% contamination. A single chromosome sequence of *Paenibacillus thiaminolyticus* NNS5-6 was deposited in the National Center for Biotechnology Information (NCBI) database under the accession number CP160395.

**Figure 6:**
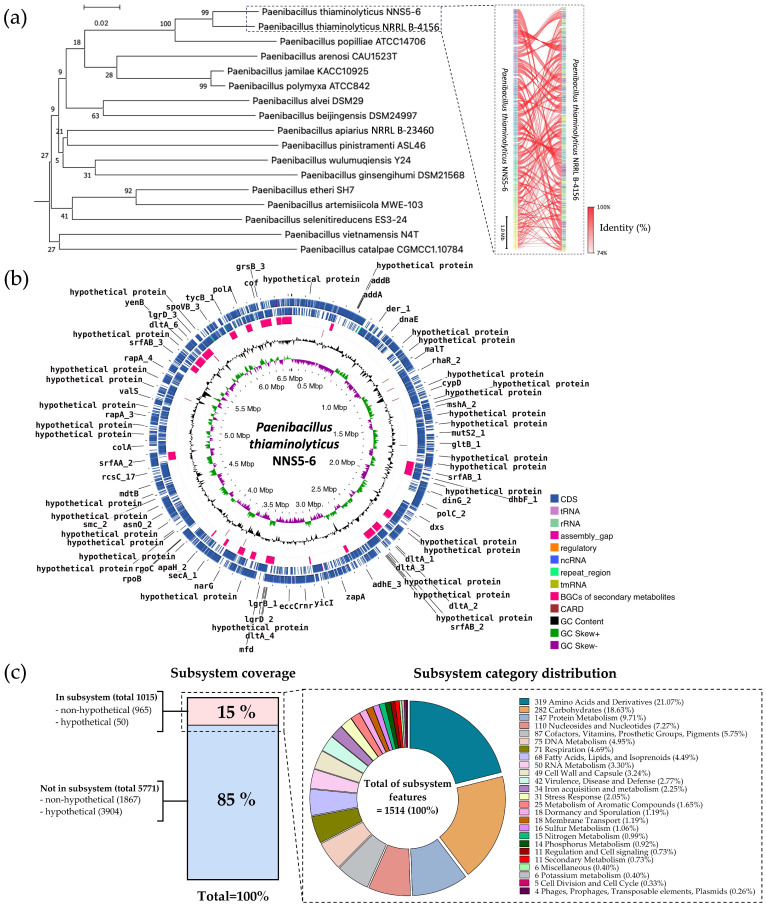
The genome-based phylogenetic tree of NNS5-6 was constructed using the GBDP method to identify the closest species and strains, via the TYGS web service. The inset visualized by FastANI demonstrates that the DNA fragments in the NNS5-6 genome are similar to the orthologous DNA fragment against the closest related genome, *Paenibacillus thiaminolyticus* NRRL B-4156 (**a**). The circular map of the NNS5-6 genome (6.5 Mb) displays the predicted coding sequences relevant to biological processes, including BGCs of secondary metabolites and antibiotic resistance genes (**b**). The subsystem technology via RAST covers 15% of the predicted subsystems, revealing the cellular machinery. The inset shows the distribution of subsystem categories from the 15% subsystem coverage, identifying 1514 features responsible for the biological processes of the bacterium (**c**).

The taxonomy of NNS5-6 was predicted using genome-based sequencing and analyzed by the Type (Strain) Genome Server (TYGS). NNS5-6 was found to show the closest relation to *Paenibacillus thiaminolyticus* NRRL B-4156. The pairwise-comparison calculated by the Genome BLAST Distance Phylogeny (GBDP) method against reference genomes in the TYGS database showed similarity scores of 74.9% (95% CI, 70.9–78.5%), 61.7% (95% CI, 58.8–64.5%), and 74.8% (95% CI, 71.3–78.0%) as d_0_, d_4_, and d_6_, respectively. The GC content of the type strain *Paenibacillus thiaminolyticus* NRRL B-4156 is 53.64%, which is similar to that of *Paenibacillus thiaminolyticus* NNS5-6 (53.20%), supporting the classification. TYGS results indicated that the NNS5-6 strain was most closely related to *Paenibacillus thiaminolyticus* NRRL B-4156 at the species level. The identity score (%) from FastANI analysis between *Paenibacillus thiaminolyticus* NRRL B-4156 and *Paenibacillus thiaminolyticus* NNS5-6 was 95.02%, supporting the GBDP results and confirming that these organisms belong to the same species. The pairwise comparison between the two species was performed by genome sequence breakdown and mapped with the fragments of orthologous DNA sequence. The similarity of each pairwise DNA fragment was visualized using FastANI v1.1.0 (Figure 6a).

The protein-coding sequences (CDS) were annotated using Rapid Prokaryotic Genome Annotation (Prokka) version 1.14.6, including biosynthetic gene clusters (BGCs) of secondary metabolites, which were annotated by Antibiotics and Secondary Metabolite Analysis Shell (antiSMASH) version 7.0. The prediction of antibiotic resistance genes localized in the genome was performed using the Resistance Gene identifier (RGI) prediction in the Comprehensive Antibiotic Resistance Database (CARD). The circular genome map and gene annotations were visualized using Proksee (CGViewBuilder version 1.1.6, https://proksee.ca, accessed on 14 July 2024), showing the number of different functions of genes, which are classified and expressed by various colors (Figure 6b). There were 6091 features annotated by Prokka, including 6024 CDS, 4 rRNA, 59 tRNA, and 4 ncRNA in the genome. The BGCs of secondary metabolites as antimicrobial compounds were counted by antiSMASH, which provided 19 BGCs with varying similarity based on orthologs in the Minimum Information about Biosynthetic Gene cluster (MIBiG) databases. The RGI prediction identified 11 possible antibiotic-resistance genes.

Rapid Annotations using Subsystems Technology (RAST) was used to predict cellular machinery based on genomic information. The genes were categorized into subsystem and non-subsystem classifications. Of the total 6786 coding sequences (CDS), 15% were categorized into subsystems and 85% into non-subsystems. The encoding proteins annotated by subsystem functionalization included 1015 genes, of which 965 genes were encoded to non-hypothetical proteins and 50 genes to hypothetical proteins. In contrast, 5771 genes annotated by familial gene evidence in the database were non-subsystem. There were 1867 genes encoding non-hypothetical proteins and 3904 genes encoding hypothetical proteins. The gene functions in the subsystem were categorized into 1514 features that ensemble for the cellular machinery (Figure 6c).

### 2.12. Comparative Analysis of Biosynthetic Gene Clusters in NNS5-6

**Figure 7:**
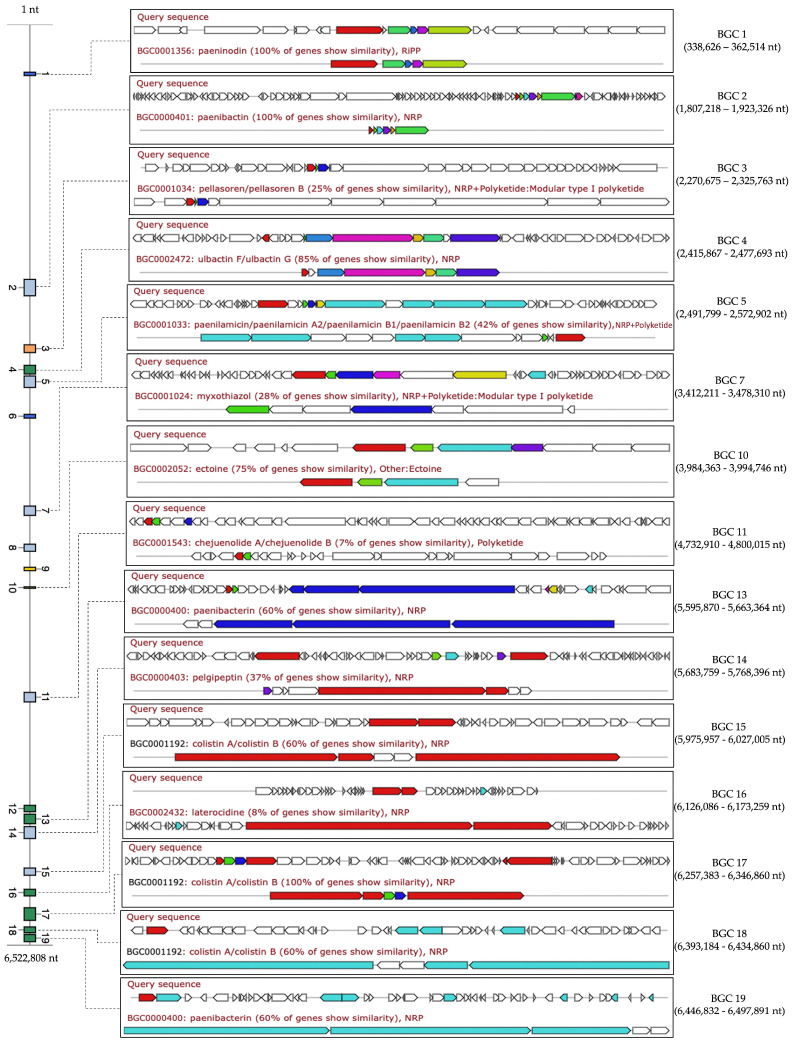
The predicted biosynthetic gene clusters (BGCs) of secondary metabolism in the NNS5-6 genome (6,522,808 nucleotides; nt) were investigated using antiSMASH. The known cluster BLAST was performed to match the reference BGCs in the MIBiG database. Nineteen BGCs were predicted and annotated for BGCs with the most similar orthologs, whereas 4 BGCs (6, 8, 9, and 12) did not match any BGCs found. The matched BGCs revealed a variety of secondary metabolites produced by the NNS5-6 genome. The color-coding of genes in the cluster followed the visualization provided by antiSMASH.

The secondary metabolites of the NNS5-6 genome were predicted using antiSMASH, which identified 19 BGCs that were thoroughly mapped in the genome. The known cluster BLAST was used to search for similar orthologs in the MIBiG database for each identified BGC, which could indicate potential secondary metabolite productions. The results from the known cluster BLAST search revealed that fifteen BGCs matched with similarities ranging from 8% to 100% based on similar orthologs, whereas four BGCs did not match any BGCs in the database. The types of secondary metabolites produced by these BGCs were categorized as non-ribosomal peptides (NRPs), ribosomally synthesized and post-translationally modified peptides (RiPPs), polyketides (PKs), co-processed production of non-ribosomal peptide and polyketide (NRP + Polyketide), and others. The identical orthologs matched to BGCs of the antimicrobial peptides with NRP and NRP + Polyketide types such as paeninodin, paenibactin, and colistin A/B exhibited 100% similarity. The high similarity of the matched orthologs ranged from 60% to 85%, including relevant BGCs for ulbactin, paenibacterin, and colistin as NRPs, while ectoin was identified as an amino acid derivative. Orthologs with less than 50% similarity could represent less explored BGCs. Among those with 8–37% similarity matched to reference BGCs in the database were polyketides (pellasoren, myxothiazole, and chejuenolide), NRPs (pelgipeptide and laterocidin), and NRP + Polyketide (paenilamicin). The matched secondary metabolites support the secondary metabolism in the Paenibacillus genus. However, BGCs 6, 8, 9, and 12 did not match any similar reference BGCs in the database, but antiSMASH predicted these types of secondary metabolites as thiopeptide, ranthipeptide, linear azol(in)e-containing peptides, and NRP, respectively (Figure 7).

**Figure 8:**
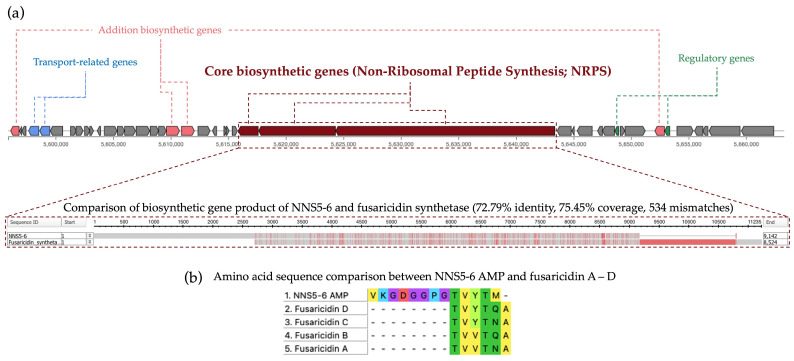
The comparative BGC of NNS5-6 AMP was proposed by the non-ribosomal peptide synthesis in antiSMASH. The BGC was composed of core biosynthetic genes, regulatory genes, and transport-related genes. The translated protein from the core biosynthetic genes showed a similar amino acid sequence to fusaricidin synthetase in the DELTA-BLAST search within the NCBI database (red and grey colors indicate different and identical amino acids, respectively). The different amino acids in the gene product comparison are indicated by red color, as visualized by the NCBI MSA viewer (**a**). The similar amino acid sequences between NNS5-6 AMP and fusaricidin A–D were compared using multiple sequence alignment with MEGA X software (version 10.1.8) (**b**).

The NNS5-6 AMP shared an amino acid sequence similar to fusaricidins, which were reported AMPs derived from Paenibacillus polymyxa. All predicted BGCs from the antiSMASH results were analyzed to find the biosynthetic gene relevant to fusaricidin production in the NNS5-6 genome. The translated protein from the core biosynthetic gene of BGC 13 (9142 amino acid residues) showed the highest similarity to fusaricidin synthetase (8524 amino acid residues; sequence ID: SUA94926.1) using the Domain Enhanced Lookup Time Accelerated BLAST (DELTA-BLAST) algorithm in the NCBI database. The result showed that the protein identity and coverage were 72.79% and 75.45%, respectively, and the mismatches were 534 amino acid residues. The comparison of translated protein sequences between the NNS5-6 core biosynthetic gene and fusaricidin synthetase showed the conserved region pattern (indicated by grey color). In contrast, the variable region contained differences in amino acid sequences (indicated by red color) (Figure 8a). The compared amino acid sequences between the two proteins support the reason that the AMP derived from NNS5-6 shares a similar amino acid sequence to fusaricidin conserved sequences (Thr-Val-Tyr-Thr) (Figure 8b). Therefore, NNS5-6 AMP could potentially be a novel AMP related to fusaricidin derivatives. However, the production mechanisms of NNS5-6 AMP and the fusaricidin-like core biosynthetic gene require further confirmation through a molecular genetic approach.

### 2.13. Prediction of Antibiotic Resistance Genes in the NNS5-6 Genome and Determination of Antibiotic Susceptibility

Assessing antimicrobial resistance is crucial when considering the use of newly discovered bacteria in various industries and healthcare sectors. To ensure safety for further utilization, it is important to investigate the NNS5-6 strain for antibiotic resistance genes and susceptibility. The whole genome sequence of NNS5-6 was analyzed for antibiotic-resistance genes using the RGI prediction in the CARD database. The analysis identified 11 predicted antibiotic-resistance genes. Among these, seven were related to glycopeptide antibiotic resistance genes with various percentages of identity and coverage when compared with reference genes in the database. Two vanY genes in the vanB cluster, two vanW genes in the vanI cluster, a vanT in the vanG cluster, a vanXY in the vanG cluster, and a vanG were identified. These predicted glycopeptide resistance genes had low percentages of identity, ranging from 33.04% to 53.47%, with 88.62% to 128.15% reference gene coverage. Additionally, other predicted antibiotic resistance genes were Otr(A) for tetracyclines, potxA for oxazolidinone antibiotics, norC for fluoroquinolone antibiotics, and qacG for antibiotic efflux pump. The identity ranged from 36.48% to 59.69%, with coverages ranging from 99.25% to 127.10% compared with the reference genes (Table 5). The antibiotic susceptibility test of NNS5-6 was conducted using the disk diffusion method to verify the predicted antibiotic resistance genes corresponding to phenotypic expression. The NNS5-6 bacterial strain exhibited high susceptibility to the combination of piperacillin (100 µg) and tazobactam (10 µg), ceftriaxone (30 µg), imipenem (10 µg), ciprofloxacin (5 µg), erythromycin (15 µg), doxycycline (30 µg), gentamicin (10 µg), cefoxitin (30 µg), and vancomycin (30 µg) (Table 6). The antibiotic susceptibility results contrasted with the predicted antibiotic resistance genes, which could be attributed to the low percentage of gene identity or reduced gene expression in the culture environment. Thus, while the resistance gene predictions provide important insights, they must be corroborated by susceptibility tests. The results indicate that NNS5-6 exhibits low resistance to commonly used antibiotics in both healthcare and industry settings.

## 3. Discussion

WHO published a critical list of pathogens requiring urgent antimicrobial development, which aimed to guide research priorities in 2017. The ESKAPE pathogens (*Enterococcus faecium, Staphylococcus aureus, Klebsiella pneumoniae, Acinetobacter baumannii, Pseudomonas aeruginosa,* and *Enterobacter* species) were designated as top priority. These bacterial species are notorious for their antibiotic resistance and present the greatest challenge in combating infectious diseases [14]. Among these, *Pseudomonas aeruginosa* and *Klebsiella pneumoniae* are of particular concern, prompting the exploration of new antimicrobial strategies.

Our research identified a novel bacterial strain, NNS5-6, from mangrove sediment samples, which exhibited strong antibacterial activity against *P. aeruginosa* TISTR 357 and *K. pneumoniae* TISTR 1383. This strain was characterized morphologically and genetically, confirming its identity as *Paenibacillus thiaminolyticus*.

The CFS of *Paenibacillus thiaminolyticus* NNS5-6 showed effective antibacterial activity against Gram-negative bacteria such as *P. aeruginosa* TISTR 357, *K. pneumoniae* TISTR 1383, and *E. coli* TISTR 887, but not against Gram-positive bacteria such as *S. aureus* TISTR 517 and MRSA strain 2468. The antibacterial components of NNS5-6 were found among the early stationary phase of the growth curve. The active components were purified using ammonium sulfate precipitation, cation-exchange chromatography, and size-exclusion chromatography. The components precipitated at 75% ammonium sulfate saturation, suggesting that the antibacterial compounds were strongly hydrophilic [15].

Our analysis identified the AMP from *Paenibacillus thiaminolyticus* NNS5-6 as having a conserved amino acid sequence similar to fusaricidins produced by *Paenibacillus polymyxa*. Fusaricidins are known for their activity against Gram-positive bacteria and fungi. Fusaricidin C and D have been reported to have similar amino acid sequences, particularly in the Thr-Val-Tyr-Thr region [16,17,18,19]. Secondary structure determination of NNS5-6 AMP, as indicated by CD studies, revealed that when dissolved in purified water or SDS micelles, the conformation of NNS5-6 AMP slightly changed, increasing the antiparallel beta-sheet and random coil structures upon contact with the lipid membrane of SDS micelles. The percentage of secondary structure proportions showed that the antiparallel beta-sheet and random coil structures were in equilibrium in both solvents. The forces governing self-assembly processes, which allow peptides to achieve a stable low-energy state, include weak interactions such as hydrogen bonding, electrostatic attractions, and Van der Waals forces. These interactions underpin the formation of secondary structures, such as alpha-helices and beta-sheets, which are crucial in all biological processes. The self-assembly processes are influenced by several factors: (i) the amino acid sequence, (ii) the degree of hydrophobicity, (iii) the length of the peptides, and (iv) the self-assembly duration. For beta-sheet structure, each beta-strand is connected laterally by hydrogen bonds, creating a pleated sheet structure that is rigidified through interpeptide and interchain hydrogen bonds. These hydrogen bonding patterns can form two different structures, parallel or antiparallel beta-sheets. Antiparallel beta-sheets are energetically more favored than parallel ones because their hydrogen bonds are better aligned [20].

NNS5-6 AMP demonstrated strong antibacterial activity with an MIC of 4 µg/mL against both *P. aeruginosa* TISTR 357 and *K. pneumoniae* TISTR 1383. Time-kill kinetics showed rapid eradication of *P. aeruginosa* TISTR 517 and *K. pneumoniae* TISTR 1383 within 8 h at 1× and 2× MIC, respectively. NNS5-6 AMP could be effective in limiting infections by these pathogens during the initial hours of bacterial colonization. Sytox Green penetration through the bacterial membrane indicates compromised cellular membrane integrity. This dye interacts with nucleic acids, leading to intense fluorescence, which reflects increased cellular permeability following the AMP action. In this study, the cell permeability test revealed that in *P. aeruginosa* TISTR 357, the AMP exhibited similar fluorescence intensity at concentrations of 2× and 1× MIC. This finding aligned with the results of time-kill kinetics, which showed comparable killing rates for 2× and 1× MIC. The AMP at a concentration of 2× MIC exhibited greater killing activity than at 1× MIC in *K. pneumoniae* TISTR 1383, which was consistent with the significantly higher fluorescence intensity observed at 2× MIC compared with 1× MIC. This study on cell permeability indicated that *P. aeruginosa* TISTR 357 exhibited greater sensitivity to NNS5-6 AMP compared with *K. pneumoniae* TISTR 1383. This observation suggests that the bactericidal effect of NNS5-6 AMP is mediated through membrane permeabilization, which correlates with the findings from time-kill kinetics and the antibacterial activity assessed using the agar well diffusion assay. However, the findings from the cell permeability experiment and the time-kill assay showed differences in the characteristics of action. This suggests that when the AMP contacts the cell membrane, it disrupts the cell membrane immediately, resulting in substantial fluorescence detection right after the addition of Sytox Green. However, the pathogen cells require sufficient time to be killed. Additionally, the differences observed in the time-kill assay and cell permeability between *P. aeruginosa* TISTR 357 and *K. pneumoniae* TISTR 1383 could be attributed to the distinct chemical compositions of their cell membranes. The outer membrane (OM) of Gram-negative bacteria is a unique structure serving as a permeability barrier against antibiotics. It consists of phospholipids, lipopolysaccharides (LPS), outer membrane beta-barrel proteins (OMP), and lipoproteins [21]. The lipid compositions of the cell membranes in *P. aeruginosa* contain 21% 1-palmitoyl-2-oleoyl-*sn*-glycero-3-phospho-(1′-*rac*-glycerol) (POPG), 60% 1-palmitoyl-2-oleoyl-*sn*-glycero-3-phosphoethanolamine (POPE), and 11% cardiolipin. The lipid compositions of the cell membrane in *K. pneumoniae* consist of 5% POPG, 82% POPE, and 6% cardiolipin. Furthermore, the lipid composition ratios of 1-palmitoyl-2-oleoyl-*sn*-glycero-3-phosphocholine (POPC):POPG: Cardiolipin in *P. aeruginosa* and *K. pneumoniae* was 1:9:1 and 7:3:1, respectively [22]. The reasons and mechanisms underlying why *P. aeruginosa* TISTR 357 is more sensitive to NNS5-6 AMP than *K. pneumoniae* TISTR 1383 requires further investigation. It is proposed that this sensitivity could be attributable to differences in the interaction between the AMP and the cell membranes of *P. aeruginosa* and *K. pneumoniae*. Therefore, the approach to determining the mechanism of action of the AMP is to investigate the AMP–membrane interaction, as bacterial membranes consist of various types of lipids and proteins [23]. Negative GRAVY values indicate a hydrophilic peptide [24]. This finding correlated with the results of NNS5-6 AMP, which has a GRAVY value of −0.231. The NNS5-6 AMP was composed of hydrophilic amino acids such as Lys, Asp, Thr, and Tyr. Hydrophobicity and hydrophilicity are crucial for understanding peptide–membrane interactions, which play a key role in permeation through target cells. Typically, highly hydrophobic peptides are more likely to form pores, whereas more polar peptides tend to interact with the negative charges on membranes. The ratio of polar to non-polar amino acids determines the interaction mechanism, thereby affecting the ability of the peptides to cross the membrane [24,25]. The positive charge of NNS5-6 AMP, containing lysine, could bind to the negatively charged bacterial membranes via electrostatic interactions. Subsequently, the hydrophobic side chains insert into the lipid bilayer, causing membrane disruption [10]. AMPs generally exert their activity by interacting with and disrupting cell membranes. This disruption can occur through several mechanisms, including pore formation (either barrel–stave or toroidal) or a carpet-like action. The secondary structure of NNS5-6 AMP was an antiparallel beta-sheet conformation, which plays an important role in forming pores in bacterial cell membranes [26]. Nevertheless, the structure–activity relationship of beta-sheet structures has been less studied than alpha-helix structures. SEM studies revealed that NNS5-6 AMP affected the surface morphology and membrane integrity of *P. aeruginosa* TISTR 357 and *K. pneumoniae* TISTR 1383. The bactericidal mechanism of NNS5-6 AMP differed based on the bacterial species. Consequently, the effect of NNS5-6 AMP on bacterial cell morphology differed distinctly between the two tested species. This variation could be attributed to differences in bacterial cell membrane compositions [27]. After treatment with NNS5-6 AMP, *P. aeruginosa* TISTR 357 cells tended to shrink and displayed noticeable holes, while *K. pneumoniae* TISTR 1383 showed signs of cell rupture with pore formation and leakage of cytoplasmic contents. *P. aeruginosa* TISTR 357 and *K. pneumoniae* TISTR 1383 cells treated with NNS5-6 AMP showed distinct characteristics to those observed in cells treated with colistin. However, these SEM results suggest the mechanisms by which the antibacterial compound interacts with the membrane.

The AMP of *Paenibacillus thiaminolyticus* NNS5-6 was sensitive to proteinase K and trypsin, confirming its proteinaceous nature. The AMP activity was found to be stable at temperatures below 40 °C. The AMP was also stable across a wide pH range (pH 4.5–10) compared with extremely acidic and alkaline environments. This information could help inform temperature conditions and buffer choices during purification and guide the formulation development of drug delivery systems for suitable administration routes. Previous research has demonstrated that fusaricidin A, produced by *Paenibacillus bovis* sp. nov BD3526, exhibits good heat stability even at 121 °C for 15 min. These compounds retain their antimicrobial activity across a broad pH range (2.0 to 9.0) and are insensitive to protease treatment [28]. The findings of this report differ from our observations regarding the fusaricidin-like AMP derived from *Paenibacillus thiaminolyticus* NNS5-6.

Phylogenetic analysis indicated that NNS5-6 had the closest taxonomic relation to *Paenibacillus thiaminolyticus* NRRL B-4156 based on genome comparison. *Paenibacillus thiaminolyticus* NRRL B-4156 was isolated from a high salinity area (5% *w*/*v* NaCl) in Japan and had a single linear chromosome with a size of 6,537,496 bp (accession number: NDGK00000000.1), 53.64% GC content, 5756 CDS, and 15 BGCs of secondary metabolisms [29]. The investigating strain, NNS5-6, showed a genome composition similar to *Paenibacillus thiaminolyticus* NRRL B-4156. *Paenibacillus* belongs to the genus within the family Paenibacillaceae. The genus is known for its aerobic or facultatively anaerobic, rod-shaped, endospore-forming, Gram-positive bacilli [30]. *Paenibacillus thiaminolyticus* was initially included in the genus *Bacillus* and was later reclassified into the genus *Paenibacillus* based on the results of 16S rRNA gene and cellular fatty acid composition analyses [31]. The vegetative cells and endospores of *Paenibacillus thiaminolyticus* NNS5-6 resembled those of *Paenibacillus thiaminolyticus* NRRL B-4156. The size of *Paenibacillus thiaminolyticus* NNS5-6 was similar to that of *Paenibacillus thiaminolyticus* NRRL B-4156 [29]. It has been reported that *Paenibacillus* species had the ability to synthesize a variety of antimicrobial compounds, including antimicrobial peptides. *Paenibacillus polymyxa* OSY-DF exhibits a promising broad spectrum of antimicrobial activity [32]. The strain produces polymyxin E1 and paenibacillin, which belong to the group of lantibiotics [33]. The antimicrobial lipopeptide, paenibacterin, derived from a soil isolate, *Paenibacillus* OSY-SE, exhibits antibacterial activity against Gram-positive and Gram-negative bacteria. Paenibacterin is a cyclic lipopeptide consisting of 13 amino acids and a C15 fatty acyl moiety [34]. There have been reports that *Paenibacillus* sp. produces polymyxins. *Paenibacillus thiaminolyticus* SY20 has been reported to produce polymyxin A1 [15]. Antimicrobial assays showed that they could inhibit numerous Gram-negative species, including *Escherichia coli* ATCC 25922, *Salmonella enteritidis* CCTCC AB 94018, *Klebsiella pneumoniae* ATCC 10031, *Enterobacter sakazakii* ATCC 29544, *Vibrio parahaemolyticus* ATCC 10031, *Psychrobacter pulmonis*, *Pseudomonas aeruginosa* PAO1, and *Salmonella typhimurium* ATCC 14028. However, the compound was ineffective against Gram-positive bacteria. On the other hand, one of the polymyxins of *Paenibacillus* sp. strain B2 was reported to be active against Gram-positive bacteria, which might be related to the presence of the unusual amino acid [35]. The predicted BGCs of the NNS5-6 genome showed paeninodin, paenibacterin, and polymyxin biosynthesis, which were consistent with the secondary metabolites reported in the *Paenibacillus* genus. *Paenibacillus thiaminolyticus* NRRL B-4156 has some BGCs similar to the NNS5-6 genome. There is a report of a fusaricidin A synthase enzyme produced by *Paenibacillus thiaminolyticus* (sequence ID: SUA94926.1) in the NCBI database. The NNS5-6 AMP is active against Gram-negative bacteria; this finding was not in accordance with previous reports of fusaricidin from *Bacillus polymyxa* KT-8, which exhibited antibacterial activity against *S. aureus* but not against Gram-negative bacteria [16]. However, a novel cyclic lipopeptide analog of fusaricidin has demonstrated antibacterial activity against Gram-negative bacteria, such as *P. aeruginosa*. The addition of positively charged exocyclic residue, such as diaminobutyric acid, has been shown to enhance activity against Gram-negative bacterial strains [36]. The predicted BGC that appeared to be relevant to NNS5-6 AMP showed high similarity to the fusaricidin synthetase found in the *Paenibacillus thiaminolyticus* NCTC11027 (accession number: UGRZ01000003.1). The fusaricidin-like core biosynthesis gene found in the *Paenibacillus thiaminolyticus* NNS5-6 genome contained a different amino acid sequence, which could be responsible for the different synthesis of the NNS5-6 AMP structure. The antibacterial effect against Gram-negative bacteria of the fusaricidin-like peptide of *Paenibacillus thiaminolyticus* NNS5-6 is hypothesized to be related to the additional amino acid sequence of the NNS5-6 AMP and could potentially alter the typical activity spectrum of the peptide [37]. The exact mechanism of action of fusaricidins and their analogs remains unclear. Studies on cyclic lipopeptides derived from the fusaricidin family have shown that they can depolarize the cytoplasmic membranes of Gram-positive bacteria in a concentration-dependent manner. However, membrane depolarization does not necessarily correlate with bacterial cell death, suggesting that membrane-targeting activity may not be the primary mode of action for fusaricidins [38]. Other research has shown that fusaricidin inhibits purine and pyrimidine synthesis. Fusaricidin treatment leads to increased degradation of nucleotide precursors, suggesting that this can reduce the availability of nucleic acid-related substances in *Bacillus subtilis*. Moreover, fusaricidin causes membrane destruction. An increase in OH production interferes with protein and nucleic acid biosynthesis in the cells [39]. Genome annotation of *Paenibacillus thiaminolyticus* NNS5-6 against the CARD database using the RGI feature revealed 11 antibiotic resistance genes, conferring resistance to four classes of antibiotics, with a low percentage of identity to the reference genes. These included glycopeptide antibiotic resistance genes, fluoroquinolone antibiotic resistance genes, tetracycline antibiotic resistance genes, oxazolidinone antibiotic resistance genes, and antibiotic efflux pump genes. The results were relevant to antibiotic-resistance genes found in *Paenibacillus thiaminolyticus* PATH554 [40]. However, antibiotic susceptibility test of *Paenibacillus thiaminolyticus* NNS5-6 verified that this strain was susceptible to commonly used antibiotics, including carbapenems, macrolides, glycopeptides, penicillins, tetracyclines, fluoroquinolones, cephalosporins, and aminoglycosides. This verification indicated that the antibiotic resistance genes identified in the RGI results showed low identity or were present at low expression levels, affecting the translation of antibiotic resistance gene products.

Although NNS5-6 AMP shows potential for killing Gram-negative bacterial pathogens through membrane disruption, the AMP would require further improvement to enhance its stability through structure optimization and delivery systems. These enhancements would assist in the successful delivery to the target site and prevent degradation due to physiological barriers. For clinical application, the study of structure-activity relationships is mandatory. However, oral and parenteral administration requires further investigation to verify therapeutic efficacy, toxicity, pharmacokinetic and pharmacodynamic properties [41]. Future research should identify the molecular mechanisms of NNS5-6 AMP and verify the predicted genes involved in its production. Safety assessments, including cytotoxicity tests In Vitro, and toxicity evaluations in vivo are essential for its applications in the health sectors. The NNS5-6 AMP represents a valuable candidate for addressing infections caused by *P. aeruginosa* and *K. pneumoniae*, potentially offering an alternative to current treatments.

## 4. Materials and Methods

### 4.1. Sample Collection and Bacterial Isolation

Mangrove sediment was collected from Thasala district, Nakhon Si Thammarat, Thailand. The five samples were randomly collected from mangrove areas at a depth of 10–15 cm. The surface part of the sediment (1–2 cm) was removed before collecting the samples. The collected samples were then placed in clean polyethylene bags and packed into an ice box. Subsequently, ten grams of sediment were transferred into a sterile flask and diluted with 90 mL of 0.85% NaCl solution (RCI Labscan Ltd., Bangkok, Thailand). Additionally, a 10% *w*/*v* sediment suspension was prepared with deionized water. Then, the pH and salinity were measured. The sediment underwent agitation in a shaking incubator at 150 rpm for 30 min under ambient conditions, followed by an additional incubation at 60 °C for 30 min. The samples were then subjected to 10-fold serial dilutions up to 10^−6^. Each dilution (100 µL) was then spread onto Mueller Hinton (MH) agar, Zobell Marine (ZM) agar, and Starch Casein (SC) agar supplemented with 1.5% NaCl (Titan Biotech Ltd., Rajasthan, India). The plates were incubated at 30 °C for a week before being re-streaked to obtain pure isolates [42].

### 4.2. Antibacterial Screening Using the Soft Agar Overlay Method against P. aeruginosa TISTR 357

A single colony of the isolate was transferred to the same solid medium used for the initial isolation and incubated at 30 °C for 3 days. *P. aeruginosa* TISTR 357 was used as test strain. The test strain was subcultured in MH agar and incubated at 37 °C for 18 h. The suspension of *P. aeruginosa* TISTR 357 was prepared by dispersing a single colony in a 0.85% sterile NaCl solution and adjusting the turbidity to be equivalent to 0.1 optical density (OD) at 625 nm (1.5 × 10^8^ CFU/mL) using a UV-visible spectrophotometer (Genesys 20, Thermo Scientific, Waltham, MA, USA). One mL of *P. aeruginosa* TISTR 357 was uniformly mixed into 9 mL of molten 0.7% agar containing MH medium and then overlaid onto the bacterial isolate-seeded plates. The soft agar overlaid plates were incubated at 37 °C for 24 h. The antibacterial compounds-producing isolates were observed by the appearance of an inhibition zone [43].

### 4.3. Verification of Antibacterial Activity Using the Agar Well Diffusion Technique

The active isolates from the agar overlay assay were transferred into broth media identical to that used during the initial isolation. The cultures were incubated at 30 °C with shaking at 150 rpm for 18 h. The optical density of the starter culture was adjusted to a turbidity of 0.1 OD at 625 nm using a 0.85% sterile NaCl solution before transferring 1 mL of the culture into 49 mL of fresh broth. The inoculum was then incubated at 30 °C with shaking at 150 rpm for 24 h. The CFS was obtained by centrifugation at 10,000× *g* at 4 °C for 15 min and filtered through a 0.2 µm sterile cellulose acetate syringe filter. The antibacterial activity of the CFS derived from each isolate was investigated using *P. aeruginosa* TISTR 357, *K. pneumoniae* TISTR 1383, *E. coli* TISTR 887, *S. typhimurium* TISTR 1469, *V. parahaemolyticus* TISTR 1596, and *S. aureus* TISTR 517 from the Thailand Institute of Scientific and Technological Research (TISTR), Thailand. The antibiotic-resistant strain, MRSA strain 2468, was kindly provided by the medical technology laboratory at the School of the Allied Health Sciences, Walailak University, Thailand. The microbial indicators were incubated at 37 °C for 18 h on MH agar. Then, the bacterial indicators were prepared to a turbidity equivalent to 0.1 OD at 625 nm before being spread on MH agar. The CFS (100 µL) was aseptically transferred to 9 mm diameter wells, followed by an incubation period at 37 °C for 18 h. Colistin (1 µg) and vancomycin (30 µg) (Sigma-Aldrich Co., St. Louis, MO, USA) were used as a positive control, while broth media was used as a negative control. The experiment was conducted in triplicate, and the mean ± SD of the diameters of the inhibition zones was measured [44].

### 4.4. Investigation of the Production Kinetics of Antimicrobial Compounds of NNS5-6

The preculture of NNS5-6 was adjusted to an OD of 0.1 at 625 nm. It was then inoculated into 50 mL of MH broth at a 2% concentration. The mixture was placed in an incubator at 30 °C with agitation at 150 rpm for 7 days. Samples were aseptically collected at intervals of 2, 4, 6, 8, 12, 16, 20, 24, 48, 72, 96, 120, 144, and 168 h. Bacterial growth was monitored by the OD at 625 nm. The CFS was collected using the same procedure as the previous experiment. The agar well diffusion assay was conducted with bacterial pathogens as described in the previous experiment. This experiment was performed in triplicate. Statistical analysis was used to compare the antibacterial activity of CFS at each incubation time. The presence of significant differences was determined by Student’s *t*-test at a *p*-value < 0.05. The kinetic of antibacterial compounds production curve with mean ± SD was presented [45].

### 4.5. Purification of the Antimicrobial Peptide

A single colony of NNS5-6 was suspended in a 0.85% sterile NaCl solution. The bacterial suspension was adjusted to a turbidity of 0.1 OD at 625 nm. The OD-adjusted suspension was used to prepare a 2% inoculum in 200 mL of MH broth in a 1 L sterile Erlenmeyer flask. The 1 L total culture was incubated at 30 °C with shaking at 150 rpm for 20 h. The CFS was collected as described in the previous experiment. Ammonium sulfate was then added stepwise to the CFS to achieve saturation levels of 25%, 50%, and 75%. The precipitates formed at each saturation level were collected by centrifugation at 18,000× *g* at 4 °C for 15 min. The collected precipitates were then dissolved in 50 mM ammonium acetate buffer solution, pH 5.0, and desalted using a dialysis bag with a 3.5 kDa molecular weight cut-off membrane (SnakeSkin membrane, Pierce, Rockford, IL, USA). The dissolved protein was dialyzed in the same buffer solution at 4 °C for 16 h. Each dialyzed fraction was tested for antibacterial activity against *P. aeruginosa* TISTR 357 using the agar well diffusion assay. Fractions exhibiting antibacterial activity were subjected to cation-exchange chromatography using HiTrap SP column (GE Healthcare Bio-Sciences AB, Uppsala, Sweden). The samples were equilibrated with a 50 mM ammonium acetate buffer solution at pH 5.0 to allow binding to the column. A gradient elution was performed using the same buffer solution with 1 M NaCl, gradually increasing from 0% to 100% over 25 mL. The eluates, detected at 214 nm absorbance, were collected and tested for antibacterial activity. Fractions containing the antibacterial compound were then injected into a size-exclusion chromatography column (Superdex™ 75 10/300 GL, GE Healthcare Bio-Sciences AB, Uppsala, Sweden). The samples were separated by molecular sieving using a mobile phase of 50 mM ammonium acetate buffer at pH 5.0. The active compound was eluted over two column volumes (50 mL) at a flow rate of 0.5 mL/min. The eluted samples were monitored by UV absorption at 214 nm, and each 1 mL fraction was collected and evaporated using a speed vacuum concentrator (RVC 2-25 CDplus, Martin Christ, Osterode am Harz, Germany). The dried residues were reconstituted in purified water to their original fraction volumes and subsequently assayed for antibacterial activity against *P. aeruginosa* TISTR 357 using the agar well diffusion method. Active fractions were then lyophilized (Gamma 2-16 LCSplus, Martin Christ, Osterode am Harz, Germany), and their masses were determined. These dried fractions were reconstituted in sterile purified water to their pre-lyophilization volumes. The reconstituted samples were subjected to serial two-fold dilutions and evaluated for antibacterial activity against *P. aeruginosa* TISTR 357. The arbitrary activity of each active fraction was calculated by taking the final dilution showing an inhibition zone to the power of 2 and multiplying it by 10.

### 4.6. Sodium Dodecyl Sulfate-Polyacrylamide Gel Electrophoresis (SDS-PAGE) and Agar Overlay Assay

The fractions purified by size-exclusion chromatography were subjected to analysis using 15% SDS-PAGE with two sets of samples to confirm the purity of the peptide by estimating the molecular weight and determining the active protein band [46]. After electrophoresis was completed, the gel was split into two parts. One part was stained with Coomassie brilliant blue G-250 for visualization of protein bands, while the other was treated with a mixture of 25% ethanol and 5% glacial acetic acid for 1 h and then washed with purified water for 3 h. The protein-fixed gel was subsequently overlaid with soft MH agar containing *P. aeruginosa* TISTR 357 (10^6^ CFU/mL) and incubated at 37 °C for 18 h to determine the position of the inhibition zone.

### 4.7. Peptide Sequencing

Peptide sequencing was performed following previously reported methods [47]. The purified sample was analyzed using an UltiMate 3000 liquid chromatography (LC) system coupled with high-resolution mass spectrometry (MS) (Thermo Fisher Scientific Inc., Waltham, MA, USA). The peptide was dissolved in 0.1% formic acid and 1% acetonitrile before being injected into a reversed-phase UHPLC column (4.6 mm × 30 mm; C18 Hypersil Gold, Thermo Fisher Scientific Inc., Waltham, MA, USA). The sample was separated by gradient elution from buffer A (0.1% formic acid) to buffer B (0.1% formic acid in acetonitrile) over 40 min at a flow rate of 300 µL/min. The separated peptides were ionized using an electrospray ionization (ESI) source with a capillary voltage of 3.2 kV at a temperature of 300 °C. De novo sequencing was conducted to determine the peptide sequences using LC-MS data in full mass scanning mode. The MS parameters for detecting the peptide fragmentation mass were as follows: resolution of 120,000, automatic gain control (AGC) target of 1 × 10^6^, maximum injection time of 100 m s, and scanning range of *m*/*z* 400–2200. The results of the full MS scan were processed using Freestyle software (version 1.4) (Thermo Fisher Scientific Inc., Waltham, MA, USA) for peak identification. The identified peaks from the parallel reaction monitoring system were analyzed for fragmentation by the second mass spectrometry (MS2). MS2 parameters were as follows: resolution of 30,000, AGC target of 1 × 10^6^, maximum injection time of 100 ms, and isolation window of *m*/*z* 1.4. The collected mass data were used to predict the amino acid sequences using Peak Studio X (Bioinformatics Solutions Inc., Waterloo, ON, Canada). The predicted peptide sequences were included in the analysis if the ALC score was above 70%. The prediction of physicochemical parameters was performed using ProtParam on the Expasy server [48].

### 4.8. Determination of the Peptide Secondary Structure

The secondary structure of the purified AMP was determined using CD spectroscopy (JASCO Corporation, Tokyo, Japan) over a wavelength range of 190–250 nm. The purified AMP (1 mg/mL) was dissolved in purified water or 50 mM SDS to determine the native conformation and to simulate the bacterial membrane environment, respectively. CD spectra were analyzed to determine the secondary structure, and the components were calculated using the BeStSel method via a web-based service [49]. Molecular modeling of the AMP structure in a three-dimensional conformation was predicted using PEP-FOLD4 for visualizing and determining molecular surface area [50].

### 4.9. Determination of Minimum Inhibitory Concentration (MIC) and Minimum Bactericidal Concentration (MBC) of NNS5-6 AMP

The MIC and MBC of the purified AMP were determined using the broth microdilution method following the guidance provided by the Clinical and Laboratory Standards Institute (CLSI) [51]. *P. aeruginosa* TISTR 357 and *K. pneumoniae* TISTR 1383 were cultured on MH agar and incubated at 37 °C for 18 h. A single colony of the tested bacteria was suspended in 0.85% NaCl until the suspension turbidity reached an OD of 0.1 at 625 nm. The cells were then diluted to 5 × 10^6^ CFU/mL using cation-adjusted Mueller Hinton broth (CAMHB). The diluted cell suspension of 10 µL was transferred into each well of a 96-well plate with a final volume of 100 µL per well. The AMP was added to achieve final concentrations ranging from 0.125 to 64 µg/mL. Colistin was used as the positive control, while antibiotic-free samples were used as negative controls. The 96-well plate was incubated at 37 °C for 24 h. Each strain was tested in triplicate. The MIC was defined as the lowest concentration of the AMP that showed no observable growth of bacteria. Subsequently, 100 µL of each dilution was spread on MH agar and then incubated at 37 °C for 24 h. The MBC was identified as the lowest concentration at which no bacterial colony growth was observed on the agar plate.

### 4.10. Scanning Electron Microscopy (SEM) of Cells Treated with NNS5-6 AMP

The effect of NNS5-6 AMP on *P. aeruginosa* TISTR 357 and *K. pneumoniae* TISTR 1383 was assessed by examining morphological alterations under SEM. *P. aeruginosa* TISTR 357 and *K. pneumoniae* TISTR 1383 were cultured in MH broth for 18 h and subsequently centrifuged to harvest the cell pellets. The cell pellets were washed with 0.85% sterile NaCl solution. The washed cells were resuspended in CAMHB to achieve a turbidity equivalent to 0.1 OD at 625 nm. The prepared cells were diluted to a concentration of 5 × 10^5^ CFU/mL in CAMHB before being treated with NNS5-6 AMP at 1× MIC for 12 h. The sample for SEM micrography was prepared by fixing the bacterial sample in 2.5% glutaraldehyde in 0.1 M phosphate buffer pH 7.2 for 24 h before ethanol dehydration of the sample. Complete ethanol removal was achieved using a critical point drying machine (Quorum Technologies Ltd., Lewes, East Sussex, UK). The dehydrated sample was coated with gold using a sputter coater machine. The micrograph image was displayed by SEM at 20,000× magnification [52]. The morphological changes in *P. aeruginosa* TISTR 357 and *K. pneumoniae* TISTR 1383 cells treated with NNS5-6 AMP were compared with those treated with 1× MIC of colistin.

### 4.11. Time-Kill Kinetics of NNS5-6 AMP

*P. aeruginosa* TISTR 357 and *K. pneumoniae* TISTR 1383 were separately adjusted to an initial treatment cell density of 5 × 10^5^ CFU/mL with CAMHB. The experiment preparation followed the microdilution assay procedures. Briefly, the NNS5-6 AMP concentrations were prepared by diluting to 1× and 2× MIC using CAMHB, while AMP-free CAMHB served as the non-treatment condition. The reactions were carried out at 37 °C, followed by spreading the entire volume from each well in each treatment condition on MH agar at specific time intervals (0–24 h). The plates were placed in a 37 °C incubator for 24 h before counting the colonies. The trends in bacterial reduction were graphically depicted using a logarithmic scale of viable cells. The bacterial cell reduction was determined within 24 h. Each experiment was performed in triplicate on the 96-well plates [53]. The presence of significant differences (*p*-value < 0.05) was assessed by two-way ANOVA, followed by post hoc Tukey’s test for conducting multiple comparisons between treated and non-treated samples at each time interval.

### 4.12. Stability Studies of NNS-5-6 AMP

The stability of the NNS5-6 AMP under various conditions at different exposure times of 1, 6, and 12 h was examined [54]. The AMP was dissolved in sterile purified water and adjusted a final concentration to 16 µg/mL. The sensitivity of the AMP to temperatures of 37, 40, 50, 60, 80, and 100 °C, as well as autoclaving at 121 °C for 15 and 30 min was evaluated. The sensitivity of the AMP to proteolytic enzymes with different digesting characteristics was studied by incubating with a concentration of 1 mg/mL of proteinase K, trypsin, and α-chymotrypsin (Sigma-Aldrich, Warren, MI, USA). The compatibility of the AMP with surfactants was evaluated by exposing the AMP to 1% SDS or 1% Triton X-100 (AppliChem GmbH, Darmstadt, Germany). The degradation of the AMP under various pH conditions, including physiological and extended alkaline conditions, was evaluated by adjusting the pH of the solution to 1.2, 4.5, 6.8, 7.4, 8.0, 10.0, 12.0, and 14.0. After incubation, the pH of the AMP solution was neutralized to the original pH before being assessed for antibacterial activity. The antibacterial assay against *P. aeruginosa* TISTR 357 was conducted using the agar well diffusion assay in triplicate. The stability profiles are presented as the percentage of the residual activity compared with the non-treatment conditions (mean ± SD), and statistical significance was determined by the Student’s *t*-test at a *p*-value < 0.05.

### 4.13. Effect of the AMP on Cell Membrane Permeability

The overnight precultured inoculum of *P. aeruginosa* TISTR 357 and *K. pneumoniae* TISTR 1383 was collected and washed three times with sterile phosphate-buffered saline (PBS) pH 7.4 supplemented with 0.2% CAMHB as a diluent. The cell suspension was diluted with the diluent until the OD at 625 nm was equal to 0.1 (1.5 × 10^8^ CFU/mL). An aliquot of the diluted cells (100 μL) was introduced into each well and incubated with 10 μM of Sytox Green (Thermo Fisher Scientific Inc., Waltham, MA, USA) in the dark for 15 min. The AMP was diluted with sterile PBS pH 7.4 supplemented with 0.2% CAMHB, and 100 μL was added to the wells to achieve final concentrations of 0.125×, 0.25×, 0.5×, 1×, and 2× MIC. After the introduction of the AMP, the cell membrane permeability of treated bacteria was evaluated by monitoring the fluorescence intensity resulting from the penetration of Sytox Green across the compromised cell membrane and subsequently binding to nucleic acid. The fluorescence intensity was measured using a microplate reader (Thermo Scientific Inc., Waltham, MA, USA) with excitation and emission wavelengths of 504 and 523 nm, respectively. Significant differences in fluorescence intensity of overall time points were analyzed by one-way ANOVA (*p*-value < 0.05) and post hoc Tukey’s test for multiple comparisons between treated and non-treated conditions [52].

### 4.14. Characterization of Bacterial Morphology

A single colony of NNS5-6 was cultured on MH agar for 1 and 3 days to obtain vegetative cells and spores, respectively. The general appearance of colony morphology was determined under a stereo microscope (Carl Zeiss, Oberkochen, Germany). Gram staining and malachite green staining were performed to determine the vegetative cell morphology and spore-forming capability, respectively, under a light microscope at 1000× magnification (Carl Zeiss, Oberkochen, Germany). The SEM micrography (Carl Zeiss, Oberkochen, Germany) was employed to examine the bacterial and spore morphology with high-resolution images at 20,000× magnification [55,56].

### 4.15. Whole Genome Sequencing and Bioinformatic Analysis

The chromosome of NNS5-6 was extracted before sequencing by Illumina Hiseq (PE150 mode, Illumina, San Diego, CA, USA) using the service of U2Bio Co., Ltd. (Seoul, Republic of Korea). Data cleaning of raw reads and genome assembly were performed using the Galaxies Australia platform version 24.0 [57]. The quality of raw reads before and after trimming the adapters was checked by FastQC version 0.12.1 [58]. The raw reads were trimmed by Fastp version 0.23.4 to remove the adapter sequences and filter out low-quality reads and reads that had a base length below 30 bases [59]. The bacterial genome was assembled by Shovill version 1.1.0 using Velvet version 1.2.10 as the assembler [60]. The quality and genome parameters of the assembled genome were evaluated by QUAST version 5.2.0 [61]. The genome completeness and contamination in the genome sequence were assessed using CheckM version 1.0.18 [62]. The known genes were annotated and predicted using Prokka version 1.14.6, and the cellular machinery was predicted using RAST [63,64]. The prediction of BGCs of secondary metabolism of antibacterial agents was carried out using antiSMASH version 7.0 [65]. The predicted secondary metabolites from the NNS5-6 BGCs were compared with the reported reference BGCs in the database that was connected to MIBiG version 3.1 and GenBank in the NCBI database [66,67]. NNS5-6 was identified at the genus and species levels using genomic data to determine the closest taxonomic relationship. Digital DNA-DNA hybridization was performed using the GBDP method to provide the similarity of the genome-based comparison between the NNS5-6 genome and the reference genome. The genome-based phylogenetic tree was constructed using the TYGS genome server [68]. FastANI version 1.1.0 in the Proksee web-based service was used to support the phylogenetic result. The sequence identity of DNA fragments in the NNS5-6 genome was calculated using the average nucleotide identity method, comparing it to the closest species identified from the GBDP result. FastANI also provided the visualization of the position of the matched DNA fragments between the two compared genomes [69]. The insights into the BGC responsible for NNS5-6 AMP production were proposed by comparing the encoded protein of the core biosynthetic gene in the NNS5-6 genome with the conserved domains of the biosynthetic enzymes in the NCBI database. The search method was performed using the Domain Enhanced Lookup Time Accelerated BLAST (DELTA-BLAST) algorithm. The matched protein from the database and the encoded protein of the core biosynthetic gene of NNS5-6 were inspected for amino acid sequences, which were aligned using the Multiple Sequence Comparison by Log-Expectation (MUSCLE) algorithm in MEGA X software version 10.1.8 [70]. The NCBI Multiple Sequence Alignment (MSA) viewer version 1.25.0 was used to determine the similarity percentage and visualize the different amino acid sequences between the encoded protein of the core biosynthetic gene in the NNS5-6 genome and database-matched protein. The antibiotic resistance genes (ARGs) in the NNS5-6 genome were assessed to support one of the safety requirements for the future utilization of the NNS5-6 isolate. The ARGs were predicted by the RGI feature provided by the CARD database [71]. The ARGs were presented with percentages of identity and coverage compared with the matched reference ARGs in the database [72]. The NNS5-6 genomic information was visualized by the Proksee web-based service [73]. The circular genome map displayed general information, including the in-depth characterization of annotated genes and predicted biosynthetic gene clusters of secondary metabolisms. The genome map information was classified using different colored tracks to better understanding and readability.

### 4.16. Antibiotic Susceptibility Studies of NNS5-6

The susceptibility of NNS5-6 to standard antibiotics was assessed using a disc diffusion assay [74]. A single colony of 18 h-precultured NNS5-6 was adjusted to an OD of 0.1 at 625 nm before being spread onto MH agar plates. Antibiotic discs (Oxoid Ltd., Hampshire, UK) of ciprofloxacin (5 µg), piperacillin (100 µg) combined with tazobactam (10 µg), imipenem (10 µg), ceftriaxone (30 µg), cefoxitin (30 µg), doxycycline (30 µg), vancomycin (30 µg), erythromycin (15 µg), and gentamicin (10 µg) were placed on the culture-seeded agar and then incubated at 30 °C for 18 h. The susceptibility tests were conducted in triplicate. The inhibition zones were reported as the mean ± SD.

## 5. Conclusions

The AMP from mangrove-derived *Paenibacillus thiaminolyticus* NNS5-6 was effective against *P. aeruginosa* TISTR 357 and *K. pneumoniae* TISTR 1383. This novel AMP had a similar amino acid sequence to fasaricidins and exhibited potent antibacterial activity by disrupting the cell membrane. The stability profile of NNS5-6 AMP showed tolerance across a wide range of pH levels, proteolytic enzymes, and surfactants, but the temperature above 40 °C should be concerned about losing activity. Genetic analysis of the NNS5-6 genome identified BGCs responsible for various secondary metabolite productions. The high susceptibility of NNS5-6 to commonly used antibiotics could serve as preliminary safety data. Future research will explore the molecular mechanisms of NNS5-6 AMP that contribute to antibacterial activity, including verification of proposed genes responsible for secondary metabolite production. The pharmacodynamics and pharmacokinetics of NNS5-6 AMP should be further studied to evaluate the efficacy of AMP, along with the comprehensive safety assessments, which are vital requirements for its clinical applications and utilizations.

## Figures and Tables

**Table 1:** Antibacterial activities of the CFS of NNS5-6 against bacterial pathogens. The antibacterial spectrum was studied using the agar well diffusion method and compared with standard antibiotics.

Isolate	Zone of Inhibition (mm ± SD; n = 3)						
	*S. aureus* TISTR 517	MRSA Strain 2468	*E. coli* TISTR 887	*K. pneumoniae* TISTR 1383	*P. aeruginosa* TISTR 357	*S. typhimurium* TISTR 1469	*V. parahaemolyticus* TISTR 1596
NNS5-6	0.00 ± 0.00	0.00 ± 0.00	14.24 ± 0.15	13.34 ± 0.53	14.84 ± 0.15	12.70 ± 0.25	13.09 ± 0.64
Vancomycin (30 µg)	21.59 ± 0.51	21.76 ± 0.78	0.00 ± 0.00	0.00 ± 0.00	0.00 ± 0.00	0.00 ± 0.00	0.00 ± 0.00
Colistin (1 µg)	0.00 ± 0.00	0.00 ± 0.00	18.13 ± 0.88	18.81 ± 0.39	18.05 ± 0.67	20.4 ± 0.89	18.8 ± 0.76

**Table 2:** The purification balance sheet of the antibacterial components of NNS5-6. The four purification steps were used to obtain the purified compound.

Purification Procedure	Volume (mL)	Total Dried Weight (mg)	Activity (AU/mL)	Total Activity (AU)	Specific Activity (AU/mg)	Purification Factor	%Yield
Crude product	976.50	453.30	20.00	19,530.00	43.08	1.00	100.00
Salt precipitation	72.78	102.40	80.00	5822.40	56.86	1.32	29.81
Cation-exchange chromatography	42.67	37.92	80.00	3413.60	90.02	2.09	17.48
Size-exclusion chromatography	12.88	3.63	160.00	2060.80	567.40	13.17	10.55

**Table 3:** The MIC and MBC values of the purified AMP of NNS5-6 were determined using the microdilution method against two bacterial pathogens. Colistin was used as the positive control.

Active Compounds	Tested Strains	MIC (µg/mL)	MBC (µg/mL)
NNS5-6 AMP	*P. aeruginosa* TISTR 357	4	4
	*K. pneumoniae* TISTR 1383	4	8
Colistin	*P. aeruginosa* TISTR 357	1	1
	*K. pneumoniae* TISTR 1383	1	1

**Table 4:** The stability of NNS5-6 AMP against temperatures, proteolytic enzymes, surfactants, and pH treatments was evaluated. The residual activity after each treatment is reported along with the incubation times (mean ± SD; n = 3).

Conditions	% Residual Activity of NNS5-6 AMP against *P. aeruginosa* TISTR 357		
	1 h	6 h	12 h
**Effect of Temperatures**			
Non-treated NNS5-6 AMP	100.00 ± 1.23	100.00 ± 0.70	100.00 ± 0.62
37 °C	99.39 ± 1.98	99.39 ± 0.61	99.69 ± 0.95
40 °C	99.80 ± 0.94	99.19 ± 0.93	99.07 ± 0.72
50 °C	65.16 ± 3.07 *	0.00 ± 0.00 *	0.00 ± 0.00 *
60 °C	0.00 ± 0.00 *	0.00 ± 0.00 *	0.00 ± 0.00 *
80 °C	0.00 ± 0.00 *	0.00 ± 0.00 *	0.00 ± 0.00 *
100 °C	0.00 ± 0.00 *	0.00 ± 0.00 *	0.00 ± 0.00 *
121 °C, 15 psi, 15 min	0.00 ± 0.00 *		
121 °C, 15 psi, 30 min	0.00 ± 0.00 *		
**Effect of Proteolytic enzymes**			
Non-treated NNS5-6 AMP	100.00 ± 0.95	100.00 ± 0.34	100.00 ± 0.58
NNS5-6 AMP with Proteinase K (1 mg/mL)	96.08 ± 3.41 *	86.58 ± 1.22 *	83.62 ± 1.34 *
NNS5-6 AMP with Trypsin (1 mg/mL)	99.38 ± 1.99	90.47 ± 2.02 *	88.90 ± 1.46 *
NNS5-6 AMP with α-chymotrypsin (1 mg/mL)	99.38 ± 0.95	99.42 ± 1.22	98.46 ± 0.88
**Effect of Surfactants**			
Non-treated NNS5-6 AMP	100.00 ± 0.60	100.00 ± 0.93	100.00 ± 0.61
NNS5-6 AMP with 1% SDS	116.77 ± 1.80 *	122.52 ± 1.86 *	119.19 ± 1.53 *
NNS5-6 AMP with 1% Triton X-100	116.17 ± 0.60 *	120.28 ± 1.53 *	120.81 ± 1.53 *
1% SDS alone	121.76 ± 2.42 *		
1% Triton X-100 alone	126.83 ± 2.72 *		
**Effect of pH variation**			
Non-treated NNS5-6 AMP	100.00 ± 1.19	100.00 ± 1.28	100.00 ± 0.72
pH 1.2	89.62 ± 1.85 *	83.83 ± 1.72 *	81.14 ± 1.71 *
pH 4.5	97.31 ± 0.33	95.69 ± 1.75	95.62 ± 1.19
pH 6.8	97.31 ± 3.33	96.46 ± 0.57	96.00 ± 0.57
pH 7.4	98.65 ± 0.58	98.18 ± 1.15	98.48 ± 0.66
pH 8.0	99.23 ± 1.15	97.22 ± 0.98	98.48 ± 0.87
pH 10.0	98.85 ± 1.20	97.42 ± 0.82	98.10 ± 1.44
pH 12.0	94.31 ± 0.68 *	93.40 ± 0.78 *	90.10 ± 2.38 *
pH 14.0	90.77 ± 0.88 *	84.40 ± 1.15 *	79.05 ± 1.75 *

* Significance according to Student’s *t*-test at a *p*-value < 0.05 compared with non-treated NNS5-6 AMP.

**Table 5:** The prediction of antibiotic-resistance genes in the NNS5-6 genome was determined by their similarity to genetic sequences in the CARD database.

Antibiotic Resistance Gene	Antibiotic Resistance Gene Family	Resistance Mechanism	Position in Genome	Identity (%)	Coverage Length (%)
*van*Y gene in *van*B cluster	*van*Y, glycopeptide resistance gene cluster	antibiotic target alteration	291,432 to 292,277	33.04	104.85
*Otr*(A)	tetracycline-resistant ribosomal protection protein of tetracycline antibiotics	antibiotic target protection	1,037,370 to 1,039,346	45.40	99.25
*van*W gene in *van*I cluster	*van*W, glycopeptide resistance gene cluster	antibiotic target alteration	1,132,154 to 1,133,269	34.43	99.46
*qac*G	small multidrug resistance (SMR) antibiotic efflux pump	antibiotic efflux	1,236,341 to 1,236,751	42.86	127.10
*potx*A	Miscellaneous ABC-F subfamily ATP-binding cassette ribosomal protection proteins of oxazolidinones antibiotics	antibiotic target protection	1,668,816 to 1,670,789	36.48	121.22
*nor*C	major facilitator superfamily (MFS) antibiotic efflux pump of fluoroquinolone antibiotics	efflux pump complex or subunit conferring antibiotic resistance	3,712,623 to 3,714,038	59.69	101.95
*van*W gene in *van*I cluster	*van*W, glycopeptide resistance gene cluster	antibiotic target alteration	3,898,660 to 3,900,096	36.69	128.15
*van*Y gene in *van*B cluster	*van*Y, glycopeptide resistance gene cluster	antibiotic target alteration	5,289,399 to 5,290,199	33.56	99.25
*van*T gene in *van*G cluster	*van*T, glycopeptide resistance gene cluster	antibiotic target alteration	5,756,379 to 5,758,274	48.63	88.62
*van*XY gene in *van*G cluster	*van*XY, glycopeptide resistance gene cluster	antibiotic target alteration	5,758,264 to 5,759,118	43.70	111.81
*van*G	Van ligase, glycopeptide resistance gene cluster	antibiotic target alteration	5,759,115 to 5,760,176	53.47	101.15

**Table 6:** Antibiotic susceptibility test results for NNS5-6, following the CLSI M100 guidance protocol.

Antibiotics	Zone of Inhibition (mm ± SD); n = 3
Ciprofloxacin (5 µg)	36.24 ± 0.63
Piperacillin (100 µg) and Tazobactam (10 µg)	50.29 ± 0.41
Imipenem (10 µg)	38.52 ± 0.52
Ceftriaxone (30 µg)	42.50 ± 0.48
Cefoxitin (30 µg)	25.40 ± 0.36
Doxycycline (30 µg)	31.07 ± 0.73
Vancomycin (30 µg)	21.51 ± 1.25
Erythromycin (15 µg)	34.88 ± 0.73
Gentamicin (10 µg)	25.57 ± 0.32

## Data Availability

Data are contained within the article and Supplementary Materials.

## Supplementary Materials

The following supporting information can be downloaded at: https://www.mdpi.com/article/10.3390/antibiotics13090846/s1, Figure S1: The purification of NNS5-6 AMP was performed using size-exclusion chromatography. Figure S2. The physical characteristics of NNS5-6.
