# Supplementary material for: Unveiling a New Antimicrobial Peptide with Efficacy against P. aeruginosa and K. pneumoniae from Mangrove-Derived Paenibacillus thiaminolyticus NNS5-6 and Genomic Analysis
Source: Antibiotics (Basel). 2024 Sep 5;13(9):846. doi: 10.3390/antibiotics13090846 (PMC11428215; doi:10.3390/antibiotics13090846)
Supplement: Supplementary file 1 [file antibiotics-13-00846-s001.zip › antibiotics-3172862-supplementary.pdf]

Unveiling a New Antimicrobial Peptide with Efficacy Against *P. aeruginosa* and *K. pneumoniae* from Mangrove-Derived *Paenibacillus thiaminolyticus* NNS5-6 and Genomic Analysis

Namfa Sermkaew [1,2], Apichart Atipairin [1,2], Sucheewin Krobthong [3], Chanat Aonbangkhen [3,4], Yodying Yingchutrakul [5], Jumpei Uchiyama [6] and Nuttapon Songnaka [1,2,*]

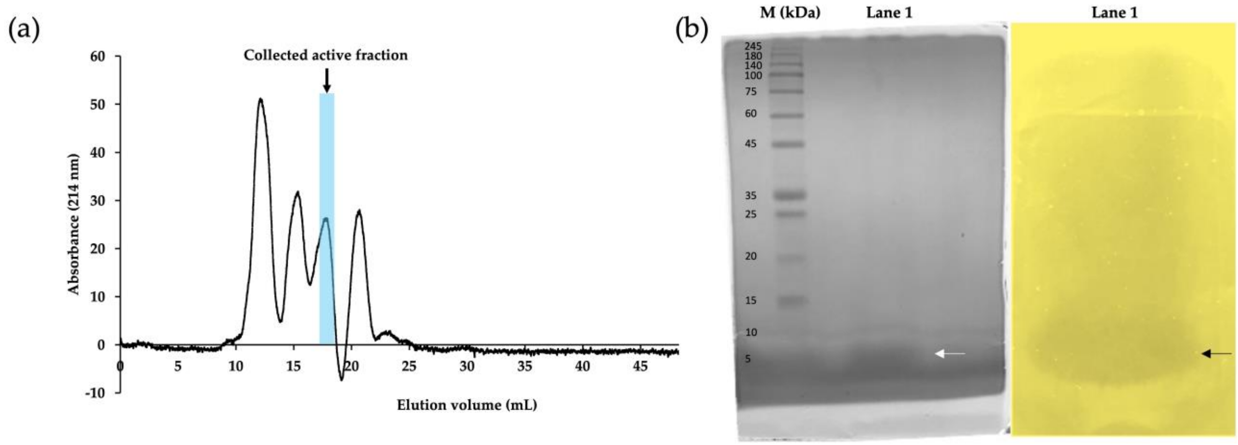

Figure S1. The purification of NNS5-6 AMP was performed using size-exclusion chromatography. The blue area indicates the collected active fractions (**a**). The peptide band of the purified AMP (white arrow) was estimated for molecular weight by SDS-PAGE, compared to a protein marker (M). The active protein band showed an inhibition zone (black arrow) as determined using soft agar overlay assay against *P. aeruginosa* TISTR 357 (Lane 1) (**b**).

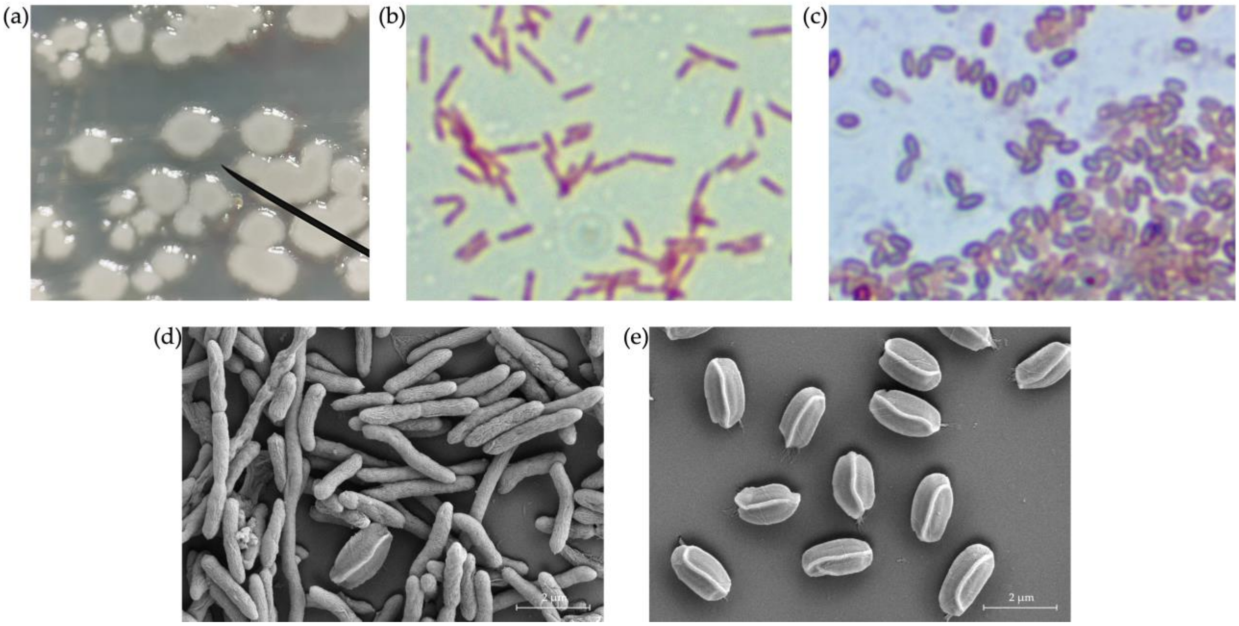

Figure S2. The physical characteristics of NNS5-6 are shown: the single colony morphology on MH agar was determined under a stereo microscope (**a**). Gram-stained vegetative cells (**b**), and malachite green-stained endospores (**c**) were observed under a light microscope at 1000× magnification. High-resolution images taken by SEM revealed vegetative cell (**d**) and endospore (**e**) morphology at 10,000× magnification.
